# Recent Developments in Bioprocessing of Recombinant Proteins: Expression Hosts and Process Development

**DOI:** 10.3389/fbioe.2019.00420

**Published:** 2019-12-20

**Authors:** Nagesh K. Tripathi, Ambuj Shrivastava

**Affiliations:** ^1^Bioprocess Scale Up Facility, Defence Research and Development Establishment, Gwalior, India; ^2^Division of Virology, Defence Research and Development Establishment, Gwalior, India

**Keywords:** protein expression, process development, upstream processing, downstream processing, high-throughput technology, perfusion cell culture, continuous chromatography, integrated continuous bioprocessing

## Abstract

Infectious diseases, along with cancers, are among the main causes of death among humans worldwide. The production of therapeutic proteins for treating diseases at large scale for millions of individuals is one of the essential needs of mankind. Recent progress in the area of recombinant DNA technologies has paved the way to producing recombinant proteins that can be used as therapeutics, vaccines, and diagnostic reagents. Recombinant proteins for these applications are mainly produced using prokaryotic and eukaryotic expression host systems such as mammalian cells, bacteria, yeast, insect cells, and transgenic plants at laboratory scale as well as in large-scale settings. The development of efficient bioprocessing strategies is crucial for industrial production of recombinant proteins of therapeutic and prophylactic importance. Recently, advances have been made in the various areas of bioprocessing and are being utilized to develop effective processes for producing recombinant proteins. These include the use of high-throughput devices for effective bioprocess optimization and of disposable systems, continuous upstream processing, continuous chromatography, integrated continuous bioprocessing, Quality by Design, and process analytical technologies to achieve quality product with higher yield. This review summarizes recent developments in the bioprocessing of recombinant proteins, including in various expression systems, bioprocess development, and the upstream and downstream processing of recombinant proteins.

## Introduction

Biopharmaceuticals are the main drugs developed in the pharma sector. Market demand has instigated the development of various protein expression hosts and bioprocessing technologies. The products approved from 2014 to mid-2018 include 68 monoclonal antibodies (mAbs), 23 hormones, 16 clotting factors, nine enzymes, and seven vaccines (Walsh, 2018). Advancements in the area of recombinant protein production have changed the previous trend, making the yield much higher and the cost much lower, thus allowing the production of such proteins on an industrial scale and opening the door for the treatment of multiple diseases and disorders. With the help of recombinant protein technology, expression of recombinant protein-based biopharmaceuticals has been achieved using bacteria, mammalian cells, yeast, insect cells, transgenic plants, and transgenic animals (Huang et al., 2012; Ahmad et al., 2014; Merlin et al., 2014; Gupta S. K. et al., 2019; Owczarek et al., 2019). *Escherichia coli* offers a fast growth rate with high product yield. Yeast systems (*Saccharomyces cerevisiae* and *Pichia pastoris*) provide post-translational modifications (PTMs). Mammalian cell lines have been used for the majority of the approved recombinant therapeutics. In the past 3–4 years, 62 of the 71 new biopharmaceutical active ingredients in the market were recombinant proteins, and of those, 52 (84%) were from mammalian cells, one from a transgenic system, five from *E. coli*, and four from *S. cerevisiae* (Walsh, 2018). A list of some recently approved recombinant biopharmaceuticals is given in Table 1.

**Table 1:** Some examples of recently approved biopharmaceuticals with their expression host systems and manufacturers/developers (Walsh, 2018).

**Product**	**Manufacturer**	**Host cell**	**Year**
Benepali (etanercept)	Samsung Bioepis	CHO cells	2016
Kovaltry (octocog alfa)	Bayer	BHK cells	2016
Rekovelle (follitropin delta)	Ferring	PER.C6 cells	2016
Alprolix (eftrenonacog alfa)	Biogen	HEK cells	2016
Inflectra (infliximab-dyyb)	Hospira	Sp2/0 cells	2016
Lartruvo (olaratumab)	Eli Lilly	NS0 cells	2016
Trogarzo (ibalizumab-uiyk)	TaiMed/ Theratechnologies	NS0 cells	2018
Taltz (ixekizumab)	Eli Lilly	CHO cells	2016
Rebinyn (rh coagulation factor IX)	Novo Nordisk	CHO cells	2017
Refixia (non-acog beta pegol)	Novo Nordisk	CHO cells	2017
Lifmior (etanercept)	Pfizer	CHO cells	2017
Truxima (rituximab)	Celltrion	CHO cells	2017
Tremfya (guselkumab)	Janssen	CHO cells	2017
Vihuma (simoctocog alfa)	Octapharma	HEK cells	2017
Adynovi (rurioctocog alfa pegol)	Baxalta	CHO cells	2018
Andexxa (coagulation factor Xa recombinant inactivated-zhzo)	Portola	CHO cells	2018
Retacrit (epoetin alfa-epbx)	Eprex and Erypo	CHO cells	2018
Shingrix (zoster vaccine)	GlaxoSmithKline	CHO cells	2018
Aimovig (erenumab-aooe)	Amgen	CHO cells	2018
Fasenra (benralizumab)	AstraZeneca	CHO cells	2018
Lamzede (velmanase alfa)	Chiesi	CHO cells	2018
Zessly (infliximab)	Sandoz	CHO cells	2018
Herzuma (trastuzumab)	Celltrion	CHO cells	2018
Fiasp (insulin aspart injection)	Novo Nordisk	*S. cerevisiae*	2017
HEPLISAV-B (hepatitis B vaccine)	Dynavax	*H. polymorpha*	2017
Semglee (insulin glargine)	Mylan	*P. pastoris*	2018
Soliqua (insulin glargine/lixisenatide)	Sanofi	*E. coli*	2016
Admelog (insulin lispro injection)	Sanofi	*E. coli*	2017
Oxervate (cenegermin-bkbj)	Dompé	*E. coli*	2017
Trumenba (meningococcal group B vaccine)	Pfizer	*E. coli*	2017
Myalepta (metreleptin)	Aegerion	*E. coli*	2018
Fulphila (pegfilgrastim-jmdb)	Mylan	*E. coli*	2018
Palynziq (pegvaliase-pqpz)	BioMarin	*E. coli*	2018
Pandemic influenza vaccine H5N1	MedImmune	Embryonated eggs	2016

After successful cell line development and clone selection, small-scale cultures using microtiter plates, test tubes, tissue culture flasks, and shake flasks are generally used for screening of recombinant protein expression. Various cultivation parameters, such as media composition, pH, agitation, aeration, temperature, cell density, the concentration of inducers, induction time, and feeding strategies affect the protein expression level depending upon expression systems (Gronemeyer et al., 2014; Tripathi, 2016). Thus, it is essential to evaluate each of the cultivation conditions for the expression of every recombinant protein and the development of effective bioprocesses. Recently, high-throughput process development (HTPD) techniques have become available and have been effectively utilized for process optimization in a cost-effective manner (Baumann and Hubbuch, 2017). Single-use upstream and downstream processing techniques have also been used for recombinant proteins to minimize the production cost and process time (Langer and Rader, 2014). After successful process development, large-scale production is carried out using bioreactor systems to fulfill the demand for biopharmaceuticals. Batch, fed-batch, and continuous or perfusion culture are used for bulk production of recombinant proteins. Continuous bioprocessing has also emerged as a novel technique and has been used in both upstream and downstream process development as well as in manufacturing for therapeutic proteins (Subramanian, 2018). The implementation of Quality by Design (QbD) and process analytical technologies (PAT) tools has improved biopharmaceutical production strategies (Kornecki and Strube, 2018; Shekhawat et al., 2019). The framework of bioprocess modeling and control also offers robust control solutions and is advantageous for optimal bioprocess design (Baumann and Hubbuch, 2017). Integrated continuous bioprocessing has also been developed recently; this allows smaller facilities and equipment footprints and facilitates rapid process development and process scale up (Godawat et al., 2015; Zydney, 2015). New developments in manufacturing processes are bringing benefits in terms of cost of production, manufacturing flexibility, and quality of the end product. The present review describes the various host systems, bioprocess development, and recent trends in bioprocessing for the production of recombinant protein-based biopharmaceuticals.

## Expression Hosts For Recombinant Protein Production

A variety of expression hosts are used for the recombinant proteins, including bacteria, mammalian cells, yeast, insect cells, transgenic animals, and transgenic plants (McKenzie and Abbott, 2018; Owczarek et al., 2019; Puetz and Wurm, 2019). Manufacturing of recombinant therapeutic proteins of high quality is crucial for their use in humans. Protein glycosylation is an important characteristic and plays a crucial role in the efficacy, serum half-life, and antigenicity of a recombinant biopharmaceutical. Expression host systems such as mammalian, yeast, and insect systems are genetically engineered to produce a human-like glycan pattern in a recombinant product to avoid side effects. Recent approaches utilized for the modification of the glycan pattern of recombinant proteins include the selection of a proper expression host, glycoengineering, and upstream process optimization to control protein glycosylation. The cell culture, biochemical, and physical process parameters are also responsible for achieving the desired glycoform of a recombinant therapeutic protein. Therefore, these parameters need to be taken into consideration carefully during the production of such glycoproteins (Gupta and Shukla, 2018). The reproducibility of the glycosylation pattern of a cell line is important to ensure product quality (Zhu, 2012). Therapeutic protein-induced antidrug antibodies can alter drug pharmacokinetics and pharmacodynamics, leading to impaired efficacy and occasionally, serious safety issues. Therapeutic protein immunogenicity risk assessment, with attention to assays and *in vivo* models, has been described as a way to mitigate this risk in a recent study (Tourdot and Hickling, 2019). The use of gene knockout/knockdown and overexpression to develop meaningful approaches to improve the PTMs of biopharmaceuticals in different production platforms and their applicability were well-described in a recent study (Amann et al., 2019). Recent developments in metabolic engineering also include the use of gene-editing tools for successful clone and product development. Innovations in cell engineering, including the use of RNAi, ribozyme engineering, and CRISPR-Cas-based techniques, have been applied in pursuit of better strategies for antibody production (Dangi et al., 2018). Gene-editing tools like CRISPR/Cas9, zinc finger nucleases (ZFNs), transcription activator-like effector nucleases (TALENs), and recombinase-mediated cassette exchange (RMCE) are being utilized for efficient gene editing (Lalonde and Durocher, 2017; Heffner et al., 2018). Genetic manipulation utilizing three major tools (CRISPR/Cas9, ZFNs, and TALENs) and associated advances have been described, with a focus on the use of CRISPR/Cas9 for the “multiplexing gene-editing approach” for genetic manipulation of yeast and CHO cells, which finally leads to rapid product development with consistency, improved product yield, quality, and affordability (Gupta and Shukla, 2017a).

### Escherichia coli

A bacterial expression host system, generally *E. coli*, is the preferable host for recombinant proteins due to its low cost, well-known biochemistry and genetics, rapid growth, and good productivity (Baeshen et al., 2015; Gupta and Shukla, 2016). Some disadvantages of this system include a lack of proper post-translational modifications (PTMs), inclusion body (IB) formation, codon bias, and endotoxin issues. Some techniques such as the addition of fusion tags (Liu M. et al., 2019) to the gene sequence, cofactor supplementation, and co-expression of the protein with molecular or chemical chaperones can avoid IB formation (Gupta S. K. et al., 2019) and improve soluble expression (Malekian et al., 2019). Different tags such as Fh8, SUMO, His, TRX, and MBP at the N- or C-terminal enhance protein solubility and also help in affinity purification (Paraskevopoulou and Falcone, 2018). Inducing protein expression by lowering the temperature after induction of culture leads to soluble protein. This strategy also increases protein stability and proper folding. Further, novel promoters and glycoengineering *E. coli* cells also lead to increased expression of a recombinant protein (Gupta and Shukla, 2016). However, the production of recombinant proteins in IBs has some advantages such as low product degradation by host cell proteases. Despite the various advantages of this system, a lack of PTM machinery results in a cumbersome purification process (Mamat et al., 2015). PTMs (glycosylation, disulfide bond formation, phosphorylation, or proteolytic processing) are involved in folding processes, stability, and biological activity (Ferrer-Miralles et al., 2009). *E. coli* has been modified for PTM for the production of recombinant proteins. An *E. coli* host has also been engineered to produce glycosylated antibodies (Wacker et al., 2002; Valderrama-Rincon et al., 2012; Gupta and Shukla, 2016). *E. coli* has been engineered to allow simple glycosylation of proteins by transferring the N-glycosylation system of *Campylobacter jejuni* into it. However, further studies are required to establish it for the industrial production of commercial therapeutic proteins (Gupta and Shukla, 2017c). The expression of genes with rare codons (e.g., those found in the human genome) often results in low expression and triggers premature termination of the synthesis of a protein molecule (Owczarek et al., 2019). The presence of rare codons can be addressed by using codon optimization or host modification. Codon optimization increases the expression of recombinant protein by many folds (Rosano and Ceccarelli, 2014; Gupta S. K. et al., 2019; Rosano et al., 2019). A redox environment and foldases [e.g., disulfide isomerases (Dsb proteins) and peptidyl-prolyl isomerases (PPIase)] are necessary to form the correct disulfide bond in the periplasm (Gupta and Shukla, 2017b). The incorporation of appropriate signal sequences for protein expression in periplasm or in the extracellular space aids correct protein folding and also minimum proteolytic degradation (Gupta and Shukla, 2016). The endotoxin problem can be solved by using a purification process to increase the safety of bacterially derived therapeutics (Mamat et al., 2015). SHuffle, an *E. coli* strain, was developed to correctly fold disulfide-bonded proteins in its cytoplasm (Lobstein et al., 2012) and was successfully used for biologically active IgG production (Robinson et al., 2015). The T7-phage polymerase, which is commonly utilized for protein expression, also results in decreased protein expression after 3–5 subsequent generations and subcultures. The novel promoter T7C p/p system can enhance recombinant protein production significantly as well as facilitating economical purification (Kesik-Brodacka et al., 2012). A specific pNEW vector has been developed using a cumate gene with a synthetic operator and the repressor protein cymR for the constitutive expression of the desired gene. This vector led to enhanced expression in comparison with a pET-based expression system (Choi et al., 2010).

MoCloFlex, a new modular cloning system for flexible *de novo* part/plasmid assembly, has been developed, and it has been revealed that it can be used to plan, build, and isolate a custom plasmid within 24 h. This leads to reduced costs and time consumption (Klein et al., 2019). In one study, it was reported that the *E. coli* “TatExpress” strain resulted in the delivery of 5.4 g/l of human growth hormone to the periplasm by the Tat pathway using fed-batch fermentation. The protein was shown to be homogeneous, disulfide-bonded, and active. Further studies are required to evaluate the full potential of this system, and especially to explore its capability for the export of more complex proteins (Guerrero Montero et al., 2019). In another study, it was shown that the use of diverse carbon and nitrogen sources and acetate metabolism knockout strains can redirect *E. coli* carbon fluxes to different pathways and resulted in a 5-fold increase in protein production (Lozano Terol et al., 2019).

Small antibody fragments such as single-chain variable fragments (scFvs) and antibody fragments (Fabs) do not need glycosylation. Therefore, these fragments have been successfully produced in *E. coli*. These antibody fragments show better tissue penetration and are less immunogenic to the human body in comparison to the full antibody. Recently, ESETEC secretion technology (Wacker Biotech) has been developed to secrete recombinant products into the culture broth during fermentation and resulted in a high yield of Fab (exceeding 4.0 g/l) and of scFv (up to 3.5 g/l) (Gupta and Shukla, 2017b). In another study, it was shown that the optimization of antibody fragment production was accompanied by the alleviation of stress production in the periplasm of *E. coli*. Thus, the monitoring of stress responses could be used to facilitate enhanced recombinant protein production yields (Baumgarten et al., 2018). Overexpression of membrane protein in *E. coli* may lead to toxicity and low yields of the active protein product. Snijder and Hakulinen (2016) described the challenges associated with overexpression of α-helical membrane proteins and different approaches to overcoming these challenges as well as a detailed protocol to express and screen membrane proteins using a His-specific fluorescent probe and fluorescent size-exclusion chromatography. Strategies for the production of soluble recombinant proteins using *E*. *coli* were described in another study (Gurramkonda et al., 2018). The latest advances in recombinant protein expression in *E. coli* were also described recently (Rosano et al., 2019).

CRISPR/Cas9 has been used to successfully carry out the chromosomal integration of large DNA into *E. coli* and was also able to integrate functional genes in diverse *E. coli* strains (Chung et al., 2017). In a recent study, it was also reported that CRISPR-Cas9-assisted native end-joining editing offered a simple strategy for efficient genetic engineering in *E. coli* (Huang et al., 2019). The existing obstacles to CRISPR-based editing in bacteria and guidelines to help achieve and enhance editing in bacteria were also described in a recent review (Vento et al., 2019). Deletion of the D-alanyl-D-alanine carboxypeptidase gene *dacC* has resulted in enhanced extracellular protein production in *E. coli* (Hu et al., 2019). Alkaline phosphatase (phoA) promoter and the heat-stable enterotoxin II (STII) leader sequence have also facilitated extracellular production in *E. coli* for the manufacture of Fab fragments (Luo et al., 2019). It was established that the post-translational targeting of single-chain variable antibody fragment (scFv) BL1 enabled its efficient production in the periplasm due to a favorable adaptation of the *E. coli* proteome (Ytterberg et al., 2019). It was also revealed that by combining signal peptide and production rate screening, enhanced recombinant protein yields were obtained in the *E. coli* periplasm (Karyolaimos et al., 2019). One study established scale-up of a type I secretion system in *E. coli* using a defined mineral medium, paving the way for industrial application (Ihling et al., 2019). The industrially important strain engineering strategies utilized to increase both the quantity and quality of therapeutic products were discussed in another study (Castiñeiras et al., 2018). Another study described the use of hierarchical-Beneficial Regulatory Targeting (h-BeReTa) employing a genome-scale metabolic model and transcriptional regulatory network (TRN) to identify the relevant TR targets for strain improvement (Koduru et al., 2018). Translating heterologous proteins places a major burden on host cells, consuming expression resources and leading to slower cell growth and productivity. In a recent study, a standard cell lysate-based assay was used to quantify the burden of expressing a protein-coding sequence and provided a parameter for predicting the burden synthetic gene expression places on *E. coli*. These lysate measurements can be utilized with a computational model of translation to predict the *in vivo* burden placed on growing *E. coli* cells for many proteins of different functions and lengths (Borkowski et al., 2018). Although a lot of work has been done on strain improvement, further technological development is still required.

### Mammalian Cells

Among all approved recombinant protein-based biopharmaceuticals, the mammalian cells dominate the other recombinant protein-expression systems (Owczarek et al., 2019). Mammalian cells have the capacity to express large and complex recombinant proteins. The introduction of the gene and selection of the clone in this system is time-consuming in comparison to microbial systems. The major steps during cell-line development include selection of an expression host, vectors, and transfection, as well as cell-line selection. High-throughput devices such as CLonePix (Thermo) and FACS (BD and Beckman) are now utilized for the development of the cell line and its screening. The major criteria for clone selection after extensive screening include a high level of protein expression with the desired PTM and genetic stability. Other features, viz. cell growth pattern, stable, and consistent production, cultivation in serum-free medium as a suspension culture, scalability in the bioreactor, adaptive performances, and product quality attributes, are also considered during clone development and selection (Gupta and Shukla, 2017c; Gupta et al., 2017). The methodologies of cell-line selection that exist for the isolation of high-producing clones and the techniques that can be utilized to predict, at a smaller scale, the performance of clones at large, industrially-relevant scales have been described in detail (Priola et al., 2016). A paper by Mauro (2018) discussed codon optimization for therapeutic protein production in mammalian cells, including potential risks and considerations.

CHO, NS0, and Sp2/0 are the main cell lines used for the expression of recombinant biopharmaceuticals. A recent survey revealed that CHO cell-based systems contribute 84% (57 of the 68 mAb products) of approved biopharmaceuticals and that the remaining antibodies are expressed in either NS0 cells (nine products) or Sp2/0 cells (two products) (Walsh, 2018). PTM is present in mammalian cell lines; however, their glycosylation pattern is different from human-type glycosylation. HEK293, HKB11, PER.C6, HeLa, and CAP cells (all human cells lines) are being also studied for protein expression (Bandaranayake and Almo, 2014; Dumont et al., 2016; Dyson, 2016; Hu et al., 2018; Gupta S. K. et al., 2019; Hunter et al., 2019). A human cell line enhances the expression of proteins with human-like PTMs. Fully glycosylated recombinant connective tissue growth factor CCN2 protein was successfully expressed using HeLa cells (Nishida et al., 2017). However, the cultivation of these cells on a commercial scale is still in the development phase. The other disadvantages of this system include contamination with animal viruses. It is difficult to formulate a culture medium for a cell line, as it requires various components such as growth factors, amino acids, reducing agents, and vitamins. CHO cells have different lineages: CHO-K1, CHO-S, CHO-DG44, and CHO-DXB11.

In a study by Reinhart et al. (2019), host cell-specific differences among CHO-K1, CHO-S, and CHO-DG44 were examined in mAb expression in batch, fed-batch, and semi-continuous perfusion cultures, revealing CHO cell line-specific preferences for mAb production. The quality attributes of mAb were also affected by the host cell line and media. It has also been established that cell engineering helps to avoid ammonium and lactate accumulation and improves cell growth (Kim and Lee, 2007). The cell line is optimized by codon optimization and various other approaches (Zhu, 2012). Further, glycoengineering is employed to produce the desired glycoform of a protein for its improved efficacy and to achieve a good-quality product (Lalonde and Durocher, 2017; Wang et al., 2017; Heffner et al., 2018). Glycoengineering strategies reduce the fucosylation or increase the sialylation of the therapeutic product. This strategy will be beneficial to industry in the future, enhancing product quality and bioactivity (Lalonde and Durocher, 2017). Tejwani et al. (2018) described in detail the advances in genetic manipulation, modeling, and glycan and glycoprotein analysis that together will present new approaches for glycoengineering of CHO cells with required or enhanced glycosylation capabilities. The types of mammalian cells used for the production of recombinant therapeutic proteins, their glycosylation potential, and the resultant impact on glycoprotein characteristics were discussed. Further, a comparison has been made between the glycosylation patterns of four recombinant glycoproteins (IgG, coagulation factor VII, erythropoietin, and alpha-1 antitrypsin) produced using different mammalian cell lines to establish the influence of mammalian host cell line on glycosylation (Goh and Ng, 2018). Strategies to increase recombinant protein expression by modulating and designing transcription factors and with advancements in synthetic biology have also been discussed (Gutiérrez-González et al., 2019). Conventional and emerging technologies for the expression of recombinant multi-protein complexes in mammalian expression systems were summarized in a review (Baser and van den Heuvel, 2016). The evolution of culture media, nutrient composition and formulation needs, optimization strategies, consistency and scalability of powder and liquid media preparation for industrial applications, and key recent advances driving progress in CHO cell culture medium design and development have been described (Ritacco et al., 2018). The major technological advancements along with the areas of application of CHO cell line development and engineering were discussed by Hong et al. (2018a). The effects of media and clonal variation on lactate shift were studied for CHO cell culture, and it was shown that the clone exhibiting lactate shift produced less lactate in the exponential phase but 2-fold higher non-toxic alanine, thus leading to a better culture environment (Hong et al., 2018b). Comparative multi-omics analysis in another study indicated some physiological variations between CHO cells grown in the same media. The protein processing abilities and the N- and O-glycosylation profiles also differed significantly across the host cell lines, suggested the necessity of choosing host cells in a rational manner for cell-line development on the basis of the recombinant protein being produced (Lakshmanan et al., 2019). A simple technique was also developed to screen multiple CHO cell clones for cell growth rate and protein production (Beketova et al., 2019). Additionally, a multi-omics study was carried out on the impact of cysteine feed level on cell viability and IgG 1 mAb production in 5 l bioreactors using CHO cells so as to obtain an in-depth understanding of the CHO cell biology (Ali et al., 2019). In a recent study, CHO cells were engineered with synthetic genetic circuits to tune the N-glycosylation of a stably expressed IgG (Chang et al., 2019).

Recent developments in metabolic engineering also include the use of knock-in (KI) and knock-out (KO) gene-editing tools for successful clone and product development. Gene-editing tools such as CRISPR/Cas9 have been successfully applied to attain better product quality for mammalian expression systems. In a recent study, C1s protease was inactivated using CRISPR/Cas9 for the production of recombinant HIV envelope protein gp120 in CHO cells (Li S. W. et al., 2019). CRISPR/Cas9-mediated site-specific integration was also used as an efficient and reliable tool for establishing recombinant stable HEK293 cell lines for biopharmaceuticals production (Yang H. et al., 2019). Through CRISPR/Cas9 gene editing, HEK293 cells were enabled to achieve antibiotic-free media bioprocessing. Further selective media and genetic optimization is required in order to increase productivity for its potential industrial use (Román et al., 2019). In another study, *Anxa2*- and *Ctsd*-knockout CHO cell lines were established by CRISPR/Cas9 and resulted in complete removal of the corresponding host cell protein (HCP) in cell lysates without affecting growth and viability for recombinant protein production (Fukuda et al., 2019). It was also reported that the CRISPR/Cas9-mediated knockout of microRNA-744 improved the antibody titer of CHO production cell lines (Raab et al., 2019). Among gene-editing tools, CRISPR/Cas9 and RMCE technologies will contribute most to the advancement of glycoprotein production in the near future.

### Yeast

Yeasts are good choices as expression hosts for recombinant proteins due to their rapid growth, easy genetic manipulation, cost-effective growth medium requirements, available complete genome sequences, and ability to provide PTMs (Fletcher et al., 2016; Vieira Gomes et al., 2018; Baghban et al., 2019; Huertas and Michán, 2019). Codon bias and extracellular expressions occur with the recombinant proteins expressed using this system. *P. pastoris* and *S. cerevisiae* are the most commonly used expression host systems for recombinant biopharmaceutical production. *S. cerevisiae* is well-established for the commercial production of therapeutics for human use. Several gene targets, most of which are involved in the trafficking and secretory pathways, that could enhance protein production by *S. cerevisiae* to the gram per liter level have been identified. It was also found that intracellular retention of recombinant proteins can be considerably reduced by engineering the endosome-to-Golgi trafficking (Huang et al., 2018). The development of a synthetic biology toolkit and how those tools have been applied in the areas of drug production and screening were described in detail by Chen et al. (2018). Due to overexpression of recombinant protein, there is intracellular accumulation, leading to reduced product titers. The hypermannosylation of proteins leads to faster blood clearance when used as therapeutics. This issue has been solved by knocking out the mannosyltransferase gene (Gupta and Shukla, 2017c). The GlycoSwitch^®^ platform has been developed and used for the production of glycosylated proteins. In it, the hypermannosylation gene (OCH1) of yeast is removed, and glycosyltransferase and glycosidase genes are introduced to produce the desired glycosylated protein (Laukens et al., 2015). However, the main issue with the above platform is the low yield of the glycosylated protein, which limits its commercial use.

*Pichia pastoris* (*a.k.a. Komagataella phaffi* or *K. pastori*) is another choice of host for heterologous protein expression due to its ability to secret properly folded and functional proteins, provide reduced protein glycosylation, and achieve high cell densities (Looser et al., 2015; Juturu and Wu, 2018; Yang and Zhang, 2018; Werten et al., 2019). However, the N-linked glycosylation patterns of this system are different in higher eukaryotes. Yeasts were genetically engineered to perform humanlike N-glycosylation (Nielsen, 2013). A study by Liu et al. (2018) reported an expressing platform and strain engineering and production processes using yeasts for antibody production, and it was concluded that the homogeneous mAb production opened a window for glycoengineering. The disadvantage of the *P. pastoris* system is the proteolytic degradation or truncation of the product, causing reduced yield, and loss of biological activity. Various strategies have been used to overcome this problem, including addition of casamino acids, yeast peptone, and protease inhibitors, optimization of induction times, reduction of pH and temperature during fermentation, and the use of alternative carbon sources (Sinha et al., 2005; Zhang et al., 2007). The establishment of systems metabolic engineering in *P. pastoris* was described in a review (Schwarzhans et al., 2017).

Overexpression of recombinant protein often leads to severe burden on the physiology of yeast and triggers cellular stress. Yu et al. (2017) identified novel factors to enhance recombinant protein production in multi-copy *K. phaffii* based on transcriptomic analysis of overexpression effects. In another study, a data-driven approach was used to analyze the secretory production of a human insulin analog precursor (IAP) in *S. cerevisiae* during prolonged cultivation (80 generations) in glucose-limited aerobic chemostat cultures. Due to long-term adaptation, a metabolic remodeling of the IAP-expressing strain was observed, leading to decreased cellular expression potential for the secretory production of IAP (Kazemi Seresht et al., 2013). In order to evaluate the potential metabolic burden that cellulase expression imposed on the yeast metabolism, two recombinant strains of *S. cerevisiae* employing two different expression strategies, namely plasmid-borne, and chromosomally expressed, were studied in comparison to a reference strain. Supplementation of the growth medium with amino acids significantly improved culture growth and enzyme production but only partially minimized the physiological effects and metabolic burden of cellulase expression (Van Rensburg et al., 2012). In another study, the problem of low secretion titers of heterologous cellulases by *S. cerevisiae* was overcome by individually over-expressing two native *S. cerevisiae* genes, PSE1 and SOD1. This overproduction of SOD1 and PSE1 genes could increase cellulase production more than 3-fold. The study demonstrated that the strain engineering can greatly improve cellulase secretion in *S. cerevisiae* (Kroukamp et al., 2013). Recently, a heterologous cellulase system was studied in *S. cerevisiae*, where two native *S. cerevisiae* genes related to yeast stress tolerance (YHB1 and SET5) were overexpressed, and their effects on the heterologous secretion of *Talaromyces emersonii* cel7A cellobiohydrolase were investigated. The recombinant strains overexpressing either YHB1 or SET5 demonstrated improved tolerance to osmotic and heat stress as well as improved heterologous secretion (Lamour et al., 2019). The applications of systems biology in *P. pastoris* range from an increased understanding of cell physiology to improving recombinant protein expression have been described (Zahrl et al., 2017). A study by Liu W. et al. (2019a) provided information on methanol metabolism during the expression of P-glycoprotein from the *P. pastoris* MutS strain and suggested an expression procedure for hard-to-express proteins from *P. pastoris*.

A novel system was reported for fast and easy expression of recombinant proteins in *S. cerevisiae* and *P. pastoris*. In *S. cerevisiae*, the gene needs only the transformation of yeast cells with an unpurified PCR product carrying the gene to be expressed, and in *P. pastoris*, it needs only the isolation of the plasmid generated in *S. cerevisiae* and its transformation into this second yeast, thus making this system suitable for HTP studies (González et al., 2018). A new, stable, autonomously replicating *P. pastoris* plasmid vector containing the full-length chromosome 2 centromeric DNA sequence was constructed that exhibits high stability for plasmid retention, facilitating genetic manipulation. This vector has the ability to speed up cloning and HTP screening in *P. pastoris*, accelerating metabolic and genome engineering and high-level protein expression in this organism (Nakamura et al., 2018). New developments related to the *P. pastoris* expression system including hosts, vectors, glycosylation pattern, and fermentation technology, as well as strain engineering using CRISPR/Cas9 technology to produce human-like glycoproteins, and protease deficient strains have been described (Baghban et al., 2018). Advances in engineering tools for *P. pastoris* including genome editing technologies for gene disruption, deletion, and editing, new chassis strains for facilitated expression of complex proteins, and innovative technologies for balanced co-expression of multiple proteins have also been described (Fischer and Glieder, 2019).

CRISPR/Cas9 was successfully applied for yeast engineering to integrate a site-specific gene or to knock out certain unwanted genes for improved recombinant biopharmaceutics production (Stovicek et al., 2015, 2017; Raschmanová et al., 2018). The recombination machinery in *P. pastoris* is less effective as compared to *S. cerevisiae*, where efficient homologous recombination naturally facilitates genetic modifications. CRISPR/Cas9 technologies for *P. pastoris* have been established and used for gene disruption studies, to introduce multiplexed gene deletions, and to test the targeted integration of homologous DNA cassettes. This system allowed rapid, marker-less genome engineering in *P. pastoris*, enabling unprecedented strain and metabolic engineering applications (Weninger et al., 2016). The CRISPR/Cas9-mediated integration of markerless donor cassettes at efficiencies approaching 100% using a *P. pastoris ku70* deletion strain was successfully demonstrated, and it was reported that the CRSIPR-Cas9 tools can be used to modify existing expression strains and provide an opportunity for markerless whole-genome modification studies in *P. pastoris* (Weninger et al., 2018). CRISPR-Cas9 was also used to develop a one-step multiloci gene integration method without the requirement of selective markers. This method can be used for pathway assembly of complicated pharmaceuticals expressed in *P. pastoris* (Liu Q. et al., 2019). The key factors that can enhance recombinant protein production in *P. pastoris* were well-described recently, and it was reported that up to 120 g DCW per liter of culture can be achieved using a chemically defined medium (García-Ortega et al., 2019). In a recent study, eight wild-type eukaryotic micro-organisms (including yeast, filamentous fungi, and mammalian cells) were evaluated to assess growth rates in industry-relevant media, adaptability for genome editing, and product quality. This study showed that multiple organisms may be suitable for recombinant protein production with appropriate engineering and development and highlighted the advantages of yeast for rapid genome engineering and development cycles (Jiang H. et al., 2019).

### Transgenic Animals

Recombinant protein-based therapeutics, including mAbs, vaccines, hormones, enzymes, and growth factors have been expressed using transgenic animals. Transgenic animals possess a transgene coding a recombinant protein that is integrated into their genome, and they are capable of passing it on to their offspring. Nowadays, the ways of sourcing proteins include milk from transgenic mammals and eggs from transgenic chickens (Moura et al., 2011; Maksimenko et al., 2013; Owczarek et al., 2019). The natural secretion of recombinant proteins occurs in this system and provides the correct PTMs. However, it is ethically questionable to produce transgenic animals. Zoonotic pathogens may be present in the protein preparations obtained from the transgenic animals (Wang et al., 2013; Bertolini et al., 2016). One study proposed potential strategies to help overcome inefficiencies in transgenic methodologies for cattle to enable the use of transgenic cattle as bioreactors for protein production in milk for industry (Monzani et al., 2016). Shepelev et al. (2018) discussed technologies for generating transgenic animals including targeted genome-editing technologies, with emphasis on the creation of animals that produce recombinant proteins in milk.

### Transgenic Plants

Transgenic plants have the ability to enhance recombinant biopharmaceutical production. This system has several advantages, viz. low cost, safety (low risk of contamination with animal pathogens), easy scale-up, stability, presence of metabolites, and ability to produce N-glycosylated proteins (Fahad et al., 2015; Yao et al., 2015; Łojewska et al., 2016; Lomonossoff and D'Aoust, 2016; Park and Wi, 2016; Xu et al., 2016; Buyel et al., 2017; Dirisala et al., 2017; Owczarek et al., 2019). Plant-based biologics have expanded to include cancer immunotherapy agents (Chen et al., 2016; Hefferon, 2017). Certain crucial factors should be considered to enhance the yield and quality of plant-produced biopharmaceuticals, namely the host plants, expression cassettes, subcellular localization, PTMs, and protein extraction and purification methodologies. DNA technology and genetic transformation methodologies have also involved to a great extent, with substantial improvements. Intensive glycoengineering study has been carried out to reduce the immunogenicity of the recombinant proteins produced in plants (Moustafa et al., 2016). The disadvantages of this system include pesticides, herbicides, and toxic plant metabolite contamination of the product. The other challenges associated with this system are control of the transgene expression level and the complex purification process. Plant cell cultures, plant tissue-based systems, and the construction of transgenic plants are mainly utilized for the production of recombinant proteins. The transgene is generally introduced into the plant cells using bacterial infection (agroinfection) or viral infection or via direct approaches such as biolistic bombardment or the PEG-mediated technique. One major advantage of these expression systems is the expression of recombinant protein in the desired cell compartment or plant organ. Human therapeutic proteins produced in plants often exhibit a plantlike rather than a humanlike glycosylation pattern. Glycoengineering is being used to solve this issue (Fischer et al., 2018; Owczarek et al., 2019). Rozov and Deineko (2019) discussed in detail the classical strategies for optimizing the synthesis of recombinant proteins and also new approaches, including gene-editing tools associated with the insertion of target genes in euchromatin genome regions.

Transgenic plants that have been used as a source of edible vaccines include rice, bananas, peas, potatoes, lettuce, and corn. A level of 100 mg/l (e.g., antibodies) or even up to 247 mg/l (e.g., α1-antitrypsin) was achieved in transgenic rice cell culture using genetic engineering (Loh et al., 2017; Owczarek et al., 2019). Human recombinant β-glucocerebrosidase (taliglucerase alfa-approved by FDA in 2012) enzyme was produced on a large scale in carrot (*Daucus carota*) cell culture (ProCellEx™) for the treatment of Gaucher disease (Tekoah et al., 2015; Moustafa et al., 2016). The world's first plant-derived IgA mAb that recognizes the surface antigen I/II of *Streptococcus mutans* (CaroRx^TM^-an anti-*S. mutans* produced in tobacco), the predominant cause dental caries, has been licensed in Europe and is used to prevent tooth decay (Larrick et al., 2001; Loh et al., 2017). Biopharmaceuticals produced in plants are at various stages of clinical trials or market implementation (Yao et al., 2015; Park and Wi, 2016; Dirisala et al., 2017; Owczarek et al., 2019). Examples are HAI-05 (Influenza Vaccine) [for Influenza A virus H5N1; host plant, tobacco (*N. tabacum*); status, phase II], Insulin (SBS-1000) [for diabetes; host plant, safflower (*Carthamus tinctorius*), status, phase III], ZMApp (monoclonal antibody cocktail) [for Ebola virus; host plant, tobacco (*N. benthamiana*); status, phase II], and Human growth hormone [for deficiency treatments; host plant, barley seed (*H. vulgare*); status, commercialization] (Owczarek et al., 2019). Human growth hormone was the first recombinant protein produced in transgenic tobacco (Barta et al., 1986; Yao et al., 2015; Loh et al., 2017).

Combined treatment of the mannosidase inhibitors kifunensine (KIF) and swainsonine (SWA) in transgenic rice cell culture media can be an effective method of producing recombinant human acid α-glucosidase (rhGAA) displaying dominantly high-mannose glycans such as Man7GlcNAc2, Man8GlcNAc2, and Man9GlcNAc2 (Man7/8/9) glycoforms without genetic manipulation of glycosylation (Choi et al., 2018). In a recent study, knockout of a green fluorescent protein (*gfp*) reporter gene in *Arabidopsis* cell culture was carried out, and it was concluded that the CRISPR/Cas9 system can be utilized for introducing site-specific mutations into the genome of cultured suspension cells of *Arabidopsis* (Permyakova et al., 2019). A new plant system based on carnivorous plants was established and showed the ability of biomimetic approaches to lead to an original production of recombinant proteins. However, the protein yields were low and did not qualify these plants for an industrial platform (Miguel et al., 2019). Recent advances in mAbs production using plant-based systems such as transgenic plants, tissue and cell cultures, and transient expression systems were described recently (Donini and Marusic, 2019). The current status of recombinant biopharmaceutical proteins generated using plant-based systems was well-documented elsewhere (Owczarek et al., 2019). A commercial-scale biotherapeutics manufacturing facility for plant-made pharmaceuticals was described by Holtz et al. (2015). Various approaches for plant-based production of recombinant proteins and recent progress in the development of plant-made therapeutics and biologics for the prevention and treatment of human diseases have also been described (Loh et al., 2017). A recent study (Rozov et al., 2018) described the similarities and differences between N- and O-glycosylation in plant and mammalian cells, as well as the effect of plant glycans on the activity, pharmacokinetics, immunity, and intensity of biosynthesis of pharmaceutical proteins. It also looked at current strategies of glycoengineering of plant expression systems to obtain fully humanized proteins for pharmaceutical application. Developments and computational tools for vaccine and antibody production in plants were also discussed recently (Dubey et al., 2018). Critical analysis of the commercial potential of plants for the production of recombinant proteins was also reported in a recent study. This study discussed the strengths of plant expression systems for specific applications, but mainly addressed the problems that must be overcome before plants can compete with conventional systems, to enable the commercial use of plants for the production of valuable proteins (Schillberg et al., 2019).

### Insect Cells

Insect cell expression host systems are also used for the expression of various recombinant proteins (Contreras-Gómez et al., 2014; Felberbaum, 2015; Kost and Kemp, 2016). The baculovirus expression vector system (BEVS) is used for the production of recombinant proteins in insect cells. The insect cells are grown to the desired cell density and then infected with a recombinant baculovirus containing the gene of interest (Owczarek et al., 2019). The glycosylation pattern in this system is comparable to, but not similar to, that of a mammalian expression system. Insect cells are not able to carry out N-glycosylation, but this issue can be solved by introducing mammalian glycosyltransferases into insect cells or by the co-expression of these enzymes together with the gene of interest in baculoviruses (Le et al., 2018). The most common cell line used for the baculovirus expression system is Sf9 (Van Oers et al., 2018; Yee et al., 2018; Ghasemi et al., 2019). In addition to Sf9 cells, S2, Sf21, Tn-368, and High-Five™ cells are also used for the expression of recombinant proteins (Contreras-Gómez et al., 2014; Felberbaum, 2015). MultiBac, an advanced baculovirus/insect cell system, has been developed and used to produce multiprotein complexes with many hitherto-inaccessible subunits for academic and industrial research and development (Sari et al., 2016; Gupta K. et al., 2019). The creation of Bac-2-the-Future, a 2nd-generation Tn7-based system, was reported, and it was demonstrated that the new system is compatible with multiple cloning methodologies and resulted in equal or better titer and protein productivity relative to the currently available systems (Mehalko and Esposito, 2016). It was also reported that a vankyrin-enhanced technology improved the baculovirus expression vector system. This study found that cell lysis could be delayed and that recombinant protein yields could be increased by using cell lines constitutively expressing vankyrin or vankyrin-encoding baculovirus vectors (Steele et al., 2017). SmartBac, a new baculovirus system, was developed for large protein complex production (Zhai et al., 2019). The FlexiBAC protein expression system was also developed for the production of both cytosolic proteins and secreted proteins that require proteolytic maturation. The design of FlexiBAC and its expansive complementary shuttle vector system enabled a reduction in cloning steps and simplification of baculovirus production (Lemaitre et al., 2019). The main methods and elements playing a role in the BEVS for protein production have been discussed in a review (Martínez-Solís et al., 2019). Many insect cell lines utilized for protein expression were found also to be persistently infected with adventitious viruses. New insect cell lines lacking adventitious viruses have been isolated for use as improved research tools and safer biological manufacturing platforms. Adventitious viruses found in insect cell lines, affected cell lines, and new virus-free cell lines were well-described in a recent review (Geisler and Jarvis, 2018).

Another study described two methods for production and purification of filovirus glycoproteins in insect and mammalian cell lines and suggested that the difficulties encountered by the authors in the purification of the proteins would facilitate other researchers to produce and purify filovirus glycoproteins rapidly (Clarke et al., 2017). The baculovirus-produced N-Terminal Pfs230 domain was also studied as a biological active transmission-blocking vaccine candidate to accelerate malaria parasite elimination (Lee et al., 2017). A baculovirus expression system was also used for the development of a combined genetic engineering vaccine for Porcine Circovirus type 2 and *Mycoplasma hyopneumoniae* (Tao et al., 2019). In one study, Hantaan virus-like particles were successfully produced by co-expressing Hantaan virus nucleocapsid (N) protein and glycoproteins (Gn and Gc) in Sf9 cells for vaccine studies, and it was shown that the purified VLPs provided protection from virus challenge in mice (Dai et al., 2019).

In a study by Mabashi-Asazuma and Jarvis (2017), various insect U6 promoters were used to construct CRISPR-Cas9 vectors, and their usefulness for site-specific genome editing in sf9 and High five cells was evaluated. This study demonstrated the use of CRISPR-Cas9 for editing an endogenous insect cell gene and altering protein glycosylation in the baculovirus-insect cell system. The successful demonstration of CRISPR in Sf9 points to a new and exciting direction for virus-less engineering of insect cells. CRISPR is expected to instigate a rapid expansion of engineering approaches to achieve enhanced expression of multiple genes in insect cells. These current and expected future developments in engineering insect cells for enhanced expression of humanized proteins are dissolving perceived disadvantages to bring about the upcoming age of the use of insect cells for the development and manufacturing of therapeutic proteins (Yee et al., 2018). Pazmiño-Ibarra et al. (2019) reported the use of a CRISPR/Cas9 system for the engineering of baculovirus to improve its performance as a protein expression vector. This study showed that the delivery of Cas9-single guide RNA ribonucleoprotein (RNP) complex with or without a DNA repair template into Sf21 insect cells through lipofection might be efficient for producing knockouts as well as knock-ins in the baculovirus (Pazmiño-Ibarra et al., 2019). A comparison of the characteristics of various expression systems used for recombinant proteins is given in Table 2.

**Table 2:** Characteristics of different expression host systems used for production of recombinant biopharmaceuticals (Demain and Vaishnav, 2009; Houdebine, 2009; Berlec and Štrukelj, 2013; Rosano and Ceccarelli, 2014; Ghag et al., 2016; Tripathi and Shrivastava, 2018; Vieira Gomes et al., 2018; Owczarek et al., 2019).

**Expression system**	**Characteristics**
Mammalian cells	Good protein folding, humanized glycosylation pattern, good secretion, slow growth rate, pyrogen-free, high overall cost, high production time, hard propagation, medium-high product yield, high product quality, very low scale-up capacity, high purification cost, high risk of contamination (virus, prions, oncogenic DNA)
*Escherichia coli*	Low overall cost, low production time, ease of cultivation, easy propagation, non-glycosylation, high growth rate, poor secretion, medium risk of contamination (endotoxins), ease of genome modifications, medium product yield, low product quality, high scale-up capacity, high purification cost, virus free
Yeast	Medium overall cost, good protein folding, glycosylation, medium production time, easy propagation, fast growth rate, high product yield, medium product quality, ease of genome modifications, good secretion, pyrogen-free, ease of cultivation, high scale-up capacity, low contamination risk, medium purification cost
Insect cells	Good protein folding, slow growth rate, high product yield, medium overall cost, feasible propagation, difficult to cultivate, medium production time, glycosylation, good secretion, medium purification cost, very low risk of contamination, high scale-up capacity, medium product quality
Transgenic plant	Good protein folding, glycosylation, very low overall cost, medium production time, very high scale-up capacity, easy propagation, high product yield, high product quality, low contamination risk, high purification cost
Transgenic animals	High overall cost, high production time, low scale-up capacity, feasible propagation, high product yield, high product quality, very high risk of contamination (virus, prions, oncogenic DNA), high purification cost

## Upstream Process Development

Innovation in bioprocessing is driven by the need for time for successful cost-effective production as well as to fulfill the demand for biopharmaceuticals. The final aim of bioprocess development is large-scale production of biopharmaceuticals. Commercial-scale process optimization is generally costly, so it is preferred to optimize processes at a small scale using laboratory bioreactors. Infectious diseases are increasing in prevalence day by day across the world, so there will be a huge demand for biopharmaceuticals. Novel concepts are being used at various stages of upstream bioprocessing, such as cell line selection and development, screening and selection of clones, optimization of media, optimization of feed, and process optimization (Shukla and Thömmes, 2010; Gronemeyer et al., 2014; Gupta and Shukla, 2017c; Gagliardi et al., 2019). During the cell development process, the selection of host cells and expression vectors and of transfection and selection methods is critical for high productivity and defined product quality (Gronemeyer et al., 2014). Process development starts with identifying cells that express the desired protein, and the identified cells are used for small scale (test tube, shake flask) and bioreactor culture to evaluate cell growth and protein production levels.

The development of an effective medium composition that includes all the essential nutrients necessary for higher cell growth and protein productivity is very important. Various commercially available cell-specific media are also used for the production of recombinant proteins. Cultivation media were previously developed using the traditional “one factor at a time” (OFAT) approach. This is essential to optimize culture medium components for every cell line individually because of cell line diversity, the medium constituents, and their interactions, processes, and metabolic pathways (Gronemeyer et al., 2014; Tripathi and Shrivastava, 2018; Gupta S. K. et al., 2019).

The batch, fed-batch, and continuous or perfusion modes of cultivation are used for the production of recombinant protein-based biopharmaceuticals (Jozala et al., 2016; Gupta and Shukla, 2017c; Tripathi and Shrivastava, 2018). In a batch mode of cultivation, all essential nutrients are provided in the initial base medium. In a fed-batch process, nutrients are fed during cultivation. In perfusion culture, the medium is circulated through a growing culture to allow simultaneous waste removal and nutrient supply (Agbogbo et al., 2019). In a continuous or chemostat bioreactor culture, feed containing essential nutrients is fed in and product containing culture is recovered continuously. If the desired rate of dilution is less than the growth rate of cells, this growth needs to be controlled using a turbidostat or chemostat culture. However, if the rate of dilution is more than the growth rate of cells, cells need to be returned back to the bioreactor (Peebo and Neubauer, 2018; Rahimi et al., 2019).

In a study by Hou et al. (2019), it was reported that the phosphorylation and hydroxylation level of an Fc-fusion protein could be reduced by nutrient optimization in a CHO fed-batch process. The application of fed-batch MTPs for HTP screening of *E. coli* clones (32 strains) was also established (Keil et al., 2019). Batch and exponential-fed-batch cultures were designed to evaluate the effect of the specific growth rate (μ) and resulted in recombinant glycoprotein AcrA glycosylation and a maximum specific synthesis rate at μ_max_ (Caillava et al., 2019). Another study evaluating continuous and fed-batch modes of cultivation for recombinant protein in *P. pastoris* revealed that at the highest μ levels and volumetric and specific productivities in the continuous mode were roughly 1.5 and 3 times greater than in the fed-batch mode (de Macedo Robert et al., 2019). The use of bioreactor technology for sustainable production of plant cell-derived products was described elsewhere (Werner et al., 2018).

For the production of therapeutic mAbs using mammalian cells, a perfusion culture is the preferred choice, because this mode decreases the residence time of the mAbs in the bioreactor. In perfusion culture, cell retention devices (tangential flow filtration, spin filters, and alternating tangential flow filtration systems) are very important for recovering culture medium containing the desired product from the bioreactor. The development and optimization of the perfusion process focuses on the transfection process, feeding strategy, cultivation time, and perfusion rate (Gronemeyer et al., 2014). In a recent study, a novel, alternative intensified cell culture perfusion process resulted in a 2-fold volumetric productivity enhancement as compared to a commercially ready, optimized fed-batch process (Gagnon et al., 2019). In another study, a single-use fluidic components-based perfusion bioreactor system was developed and enabled the implementation of active environmental control (Bournonville et al., 2019). In a study by Bertrand et al. (2019), the impact of perfusion cultivation on the intracellular physiological state of a CHO cell line was investigated, revealing decreased mAb productivity as well as a transition phase for metabolites and product quality before reaching steady-state conditions. For viral vaccine production using anchorage-dependent cells (e.g., Vero cells), microcarriers are necessary in the bioreactor. The microcarriers also provide protection to the cells from excessive shear (Hu et al., 2011; Merten, 2015).

Bioreactor type and process control are also important factors to consider for successful process optimization and effective process development (Butler and Meneses-Acosta, 2012). The bioreactors used for biopharmaceuticals production include stirred tank bioreactors, airlift bioreactors, bubble column bioreactors, hollow fiber bioreactors, and fixed bed and fluidized bed bioreactors (Warnock and Al-Rubeai, 2006; Jain and Kumar, 2008; Vermasvuori and Hurme, 2011; Rivas-Interián et al., 2019). Membrane bioreactors are also available commercially (miniPERM bioreactor from Vivascience and CELLine from Integra Biosciences) and are utilized for small-scale production of mAbs (Dewar et al., 2005).

The various operating parameters [temperature, pH, agitation, aeration, dissolved oxygen (DO), CO_2_, and hydrodynamic shear] used for bioreactor cultivation also need optimization to achieve enhanced productivity for recombinant biopharmaceuticals using different protein expression host systems. The successful development and optimization of a bioprocess also takes into account temperature shifts and gas exchange during cultivation. The optimization of all of the above-described parameters results in high cell densities and enhanced specific and volumetric productivities with better product quality. Successful process optimization strategies have resulted in an increase in product yield from 50 mg/l to 5–20 g/l for mAbs (Gronemeyer et al., 2014). In a recent study, it was also established that aeration and shear stress were critical process parameters for the production of oncolytic measles virus using Vero cells (Grein et al., 2019). In a recent review, the methodology and devices used for oxygen uptake rate determination were well-described (Martínez-Monge et al., 2019).

Upstream process development also includes scale-up of a fermentation process to ensure a similar product yield with quality at large scale as is produced at small scale. Thorough knowledge of bioreactor parameters at various scales helps the successful scale-up of robust production processes. The important parameters for scale-up, which are critical to efficient cell growth, viability, and protein production, include mixing, oxygen transfer, heat-transfer characteristics, and shear forces (Werner, 2013). The most commonly used criterion for scale-up is to keep one or more parameters similar between different scales. Such parameters include constant agitation power input (power/volume), constant oxygen transfer coefficient, constant mixing time, constant agitation impeller tip speed, constant heat transfer rate (heat/volume), constant gas volumetric flow rate (vvm), and constant gas superficial velocity (m/s) (Junker, 2004; Schmidt, 2005; Garcia-Ochoa and Gomez, 2009; Xu et al., 2017). Linear scale-up parameters include temperature, pH, pressure, DO, airflow rate, and nutrient concentrations. In general, oxygen transfer rates (OTRs) decrease as fermentor scales increase (Yang, 2010). The challenge to meet temperature control requirements may be due to the limited cooling capacity of a large bioreactor. Pressure may also be adjusted as a strategy or tool to improve gas transfer at larger scales where a high agitation rate is difficult to reach (Islam et al., 2008; Lee, 2009; Meagher et al., 2011; Zawada et al., 2011; Ruiz et al., 2013). It was also reported that understanding the genetic heterogeneity will inform metabolic engineering and synthetic biology approaches to reduce the emergence of non-producer mutants in scaled-up fermentations and increase product quality and yield (Rugbjerg and Sommer, 2019). Ultimately, successful scale-up is determined to have been achieved when comparable process performance endpoints such as cell growth, cell viability, protein production (i.e., titer), and product quality are achieved.

**Figure 1:**
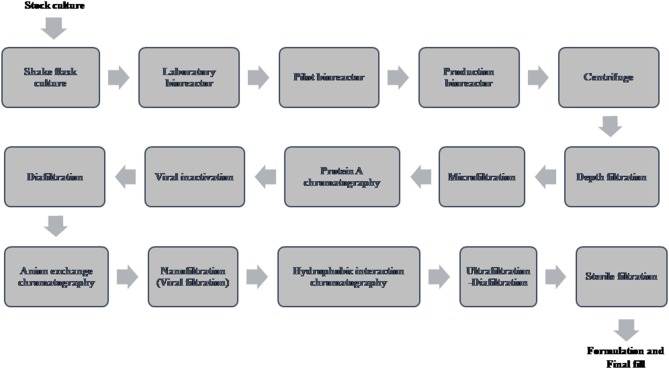
Flow chart of the production of therapeutic monoclonal antibody using mammalian cell culture.

A typical flow chart for the large-scale production of mAb using mammalian cells is shown in Figure 1. The upstream process includes inoculation of working cell stock into small-scale shake flask cultures followed by laboratory-scale and pilot-scale bioreactors and final cultivation into a production bioreactor. The downstream manufacturing process includes cell harvesting (using centrifugation and depth filtration), microfiltration, Protein A chromatography, viral inactivation, diafiltration, anion exchange chromatography (AEX), viral filtration, hydrophobic interaction (HI) or cation exchange chromatography (CEX), diafiltration, sterile filtration, and formulation.

## Recent Developments In Upstream Processing

Recent innovation in the upstream processing has resulted in cost-effective, high yield, and speedy production of recombinant protein-based biopharmaceuticals. The innovative technologies used for successful upstream process developments include high-throughput (HTP) technologies, single-use devices, statistical optimization of media and environmental parameters, QbD, PAT, and continuous upstream processing.

### High-Throughput Cultivation Systems

High-throughput devices (HTPDs) have been developed for upstream process development such as multi-well-plates and mini-bioreactors. Using these HTPDs, it is now very easy to do all the screening experiments including optimization of processes to save time and cost before proceeding to scale up the production of recombinant proteins. Examples of high-throughput systems are miniature shaken vessel/wells or microtiter plates (MTPs), bubble column or microplate-based mini-bioreactors, and stirred mini-tank bioreactors. Batch and fed-batch process optimization have been established and improved using HTPD. Process optimization for perfusion cell culture is needed for successful continuous bioprocessing. For this reason, the equipment developers and manufacturers have a motivation to develop suitable HTP perfusion microbioreactors for perfusion process optimization studies (Fisher et al., 2018). Cultivations in standard 96-well-microtiter plates represent the optimal system for miniaturization (Duetz, 2007; Baumann and Hubbuch, 2017). MTPs were successfully used for recombinant protein process development (Long et al., 2014; Chung et al., 2018; Fink et al., 2019; Keil et al., 2019).

A 10–15 ml microscale bioreactor (ambr) (Sartorius) with a fully automated robotic workstation and feeding and sampling was used for mAbs production from CHO cells. BioLector (m2p-labs) cultivation plates [with an optical bottom and optical sensors (for pH and oxygen transfer rates)] have become available and have been used for recombinant proteins. In one study, a continuous perfusion microbioreactor system (1-ml working volume) was developed and demonstrated to have a perfusion rate of 1 ml/h (Mozdzierz et al., 2015; Fisher et al., 2018). The ambr 250 was successfully demonstrated as a representative scale-down model for two mAbs commercial processes at scales of >10,000 l (Manahan et al., 2019). A polymer-based controlled-release fed-batch microtiter plate (48-well-plate) with on-line monitoring capabilities was also studied with *E. coli* for screenings and initial process development (Habicher et al., 2019a). In a study by Fink et al. (2019), 32 production clones were characterized in carbon-limited microbioreactor cultivations (BioLector), with production yields of 0–7.4 mg Fab per gram of cell dry mass. The use of polymer-based controlled-release fed-batch microtiter plates during preculture was theoretically studied and tested with an *E. coli* clone bank containing 32 strains (Keil et al., 2019). The suitability of an automated small-scale bioreactor (ambr 15 with 48 wells) as a small-scale model was confirmed using a perfusion process (Janoschek et al., 2019). In another study, it was reported that an automated microbioreactor system (ambr15) can be utilized to scale down the perfusion process using cell sedimentation (as the cell retention method). It was observed that this model under-predicted cell line productivity but accurately predicted product quality attributes, including glycosylation profiles, from cultures carried out in 1 l and 1,000 l working-volume bioreactors. The microbioreactor system allowed an 80-fold decrease in culture media requirements and halved the daily operator time, resulting in an approximately 2.5-fold cost reduction compared to a similar bench-scale experimental setup (Kreye et al., 2019). The impact of the bioreactor scale (10 ml ambr as the scale-down model and a 300 l pilot scale) on intracellular micro-heterogeneities in a CHO cell line producing mAbs in fed-batch mode was studied by Bertrand et al. (2018), revealing that the enzymatic activity was affected by the varying environmental conditions, leading to an observed time-dependent variation. In another study, FeedER (feedback-regulated enzyme-based slow-release system) exponential fed-batch for microscale cultivations was developed that enabled 48 fed-batch experiments to be run in parallel in an automated and miniaturized manner. This can significantly accelerate the bioprocess scale-up from lab scale to industrial scale. Future work will focus on the application of this system for different feeding modes, e.g., constant feed rates or different slow-release systems (Jansen et al., 2019). In another study, a fully automated microbial cultivation platform (capable of performing up to 32 fed-batch cultivations simultaneously) was developed, and it was reported that the initial performance (with respect to different expression systems and process conditions) of this platform was comparable to 5 l cultivations. Thus, fully automated HTP cultivation (with automated centralized data storage) considerably speed up the identification of the optimal expression systems and process conditions, offering the potential for automated early-stage bioprocess development (Janzen et al., 2019). A systematic analysis of HTP scale-down models (ambr^®^250, 250 ml) was carried out for 500 or 2,000 l single-use bioreactors to produce a mAb using vvm (volume of gas per volume of liquid per minute) as the scale-up criterion. This study reported that scale-down using a similar vvm as the criterion was feasible for reproducing large-scale gas transfer characteristics (Zhang et al., 2019).

In another study, hydrodynamic conditions, and mass transfer in miniaturized bubble column (MBC) bioreactors were investigated using *E. coli*. The gas hold-up and volumetric mass transfer coefficient (K_L_a) in MBCs were found up to ten times greater than those in the larger bubble columns and comparable to the stirred tank bioreactors (Khanchezar et al., 2019). A membrane-based fed-batch shake flask with a Respiration activity monitoring system (RAMOS) device was also used to study the effect of substrate-limited fed-batch conditions (Habicher et al., 2019b). A mini-chemostat (MC) system (16 reactors with 40 ml working volume) was developed to characterize yeast physiology, and it was shown that the MC system provided the same environmental conditions as the DASGIP^®^ parallel bioreactor system (Eppendorf) (Bergenholm et al., 2019). In a recent review, a systematic approach toward scale-down model (SDM) development in ambr 15 systems was described, and it was suggested that ambr SDMs are suitable for future regulatory submissions (Sandner et al., 2018). In a study by Vit et al. (2019), the efficiency of a microfluidic cell cultivation device and its applicability for rapid screening of multiple parameters was also established. A list of some HTP devices is given in Table 3.

**Table 3:** List of some HTPD systems used for upstream process development (Baumann and Hubbuch, 2017).

**System details**	**Manufacturer/developer**
ambr 15 microbioreactor (24- or 48-reactor, STR), 10–15 ml	Sartorius
ambr 250 microbioreactor (12- or 24-reactor, STR), 100–250 ml	
BioLector (48-reactor, MTP), 800–2,400 μl	m2p-labs
BioLevitator (4-reactor, tube), 50 ml	Hamilton Bonaduz
bioREACTOR (48-reactor, STR), 8–15 ml	2 mag AG
bioREACTOR (8-reactor, STR), 8–15 ml	
DASbox mini bioreactor (24-reactor, STR), 60–250 ml	Eppendorf
Micro-24 MicroReactor system (24-reactor, MTP), 3–7 ml	Pall
Micro-Flask (24- or 96-square deep well-plates), 0.5–4 ml	Applikon
Micro-Flask (24- or 96-round low well-plates), 0.1–1 ml	
Micro-Matrix (24-reactor, MTP), 1–5 ml	

### Single-Use Cultivation Technologies

Recent progress in single-use (SU) cultivation systems, including single-use probes/sensors and fluidics components, has led to rapid developments of upstream processing. The implementation of single-use upstream processing devices resulted in less capital and operating costs with greater flexibility (Boedeker et al., 2017). A study on integrated continuous processing reported that cost savings of about 30% can be achieved using disposable technologies with respect to stainless steel (SS) batch process (Jacquemart et al., 2016; Hummel et al., 2018; Somasundaram et al., 2018). The different varieties of single-use disposable cultivation systems include wave, orbital shaken (OS), stirred tank (ST), and pneumatically mixed bioreactors (Shukla and Gottschalk, 2013; Raven et al., 2015; Challener, 2017; Ghasemi et al., 2019). There is very low risk of contamination with these systems, as cleaning and sterilization are not needed. A list of some single-use systems is given in Table 4.

**Table 4:** List of some single-use systems used for upstream process development (Gupta S. K. et al., 2019).

**System details**	**Manufacturer/developer**
DASboxMini bioreactor system, 60–250 ml	Eppendorf
DASGIP parallel bioreactor system, 320 ml−3.75 l	
CelliGen BLU, 5–50 l	
Applifex systems, 500 ml	Applikon
Xcellerex XDR (STR), 50–2,000 l	GE
Wave bioreactor system, 0.1–500 l	
ambr 15 (24 or 48 microbioreactor), 10–15 ml	Sartorius
BIOSTAT STR, 2.5–2,000 l	
Micro-24 MicroReactor, 3–7 ml system	Pall
CELL-tainer, 250 ml−200 l	Celltainer Biotech
Mobius CellReady, 3 l	Merck Millipore
HyPerforma single-use bioreactors, 30–300 l	Thermo scientific

A wave bioreactor system (GE) has been developed that consists of a Cellbag (made of polymers) placed on a rocker unit equipped with controllers for pH, DO, temperature, and pressure. This design improves the mixing of the cultivation media and mass transfer (Ghasemi et al., 2019). Some disadvantages associated with a bag-based bioreactor are the risk of leaching from the plastic bag to the product and decreased process performance due to binding of media constituents with plastic (Shukla and Gottschalk, 2013; Gupta and Shukla, 2017c). Disposable Wave bioreactors up to a 500 l scale, disposable ST bioreactors up to a 2,000 l scale (Xcellerex XDR 2000; GE), and orbitally shaken (OS) bioreactors (with a cylindrical or square-shaped vessel) up to a 2,500 l scale (De Jesus and Wurm, 2011) are available and are used for cultivations. The single-use Biostat B (1 l and 5 l bioreactors) and single-use ST bioreactor (Mobius 3 l) are also available and are utilized in process development (Gupta S. K. et al., 2019). The operating cost is high at large scale due to frequent purchasing of new bags. Single-use cultivation systems are also utilized to prepare inoculum for large-scale cultivations (Mahajan et al., 2010).

In one study, scale-up was carried out in a 200 l disposable OS bioreactor with BY-2 cells (Tobacco) for the production of the human mAb M12 and resulted in 300–387 g/l cell fresh weights with ~20 mg/l M12 (Raven et al., 2015). In another study, a Wave bioreactor at the 2 l scale was used to cultivate Sf9 cells and infected/non-infected BTI-TN-5B1-4 cells to estimate the specific oxygen uptake rates. Using these results, active soluble human papillomavirus (HPV) 16L1 protein expression was scaled up to 10 l and 50 l cell bags, resulting in a 10% decrease in volumetric protein expression (Ghasemi et al., 2019).

Single-use bioreactor (microcarrier-based) culture is a good option for viral vectors and viral vaccines. A microcarrier bead-to-bead expansion and transfer process was established for HEK293T cells and Vero cells and scaled up to 50–200 l using XDR-50 and XDR-200 bioreactors, resulting in 3.3 × 10^6^ cells/ml in the XDR-200 bioreactor with Vero cells (Yang J. et al., 2019).

### Design of Experiments Approach

In order to minimize experimental effort in upstream process development, the Design of Experiments (DoE) approach has also been applied to investigate the various process parameters in recombinant protein production (Papaneophytou and Kontopidis, 2012; Hanke and Ottens, 2014; Shekhawat et al., 2019). Statistical experiments using various DoE approaches, such as full factorial design, fractional factorial design, Taguchi orthogonal arrays, and the response surface methodology (RSM), were used for the optimization of media to enhance protein yield because various components are present in media that interact with each other. In DOE, various process parameters can be changed in a set of experimental trials, and a small number of experiments are enough to decide the effect of the various parameters and to select the most important ones (Papaneophytou and Kontopidis, 2014; Kumar et al., 2019; Shekhawat et al., 2019). In one study, RSM was applied to develop a defined medium to enhance human interferon gamma production (Unni et al., 2019). Using DoE, the signal peptide was selected and optimal growth conditions were established for recombinant antibody fragment production in the periplasm of *E. coli* (Kasli et al., 2019).

### Quality by Design (QbD) Approach

The concepts of Quality by Design (QbD) with high-throughput devices or Design of Experiments have also been studied for upstream process development. QbD is a manufacturing principle in which product quality is integrated into the manufacturing process. The QbD method has been implemented for the process development and analytical characterization of recombinant proteins, including mAbs (Pathak et al., 2014; Yu et al., 2014; Kumar et al., 2019; Narayanan et al., 2019a; Shekhawat et al., 2019). Using the QbD approach, the effect of various media components and process parameters in Fab production was studied, resulting in a 5-fold enhancement of the target protein titer as compared to the basal medium, thus demonstrating the efficacy of QbD (Kumar et al., 2019).

### Process Analytical Technology (PAT) for Upstream Processes

Effective process optimization requires real-time monitoring of different process parameters. PAT is the process of ensuring that final product quality meets specifications by designing, analyzing, and controlling manufacturing through periodic and/or continuous measurement of critical quality and performance attributes. Critical quality attributes (CQAs) are properties that ensure the desired product quality by meeting defined criteria. Process parameters that affect CQA are called critical process parameters and need to be observed or controlled to ensure that the process leads to the desired quality. There is a need for innovation in sensor technology, its configuration, and its robustness so that PAT can be implemented for the advancement of continuous cultures (Fisher et al., 2018). The development of process analytical tools for analysis of the performance of perfusion bioreactor cultures has made significant contributions in terms of regulatory issues regarding the manufacturing of proteins (Somasundaram et al., 2018). The PAT tools based on spectrometry, which are used for on-line (integrated into the bioreactor system, i.e., lying outside the bioreactor, requiring an automatic sampling interface to the bioreactor that enables a sample to be drawn and delivered to the analyzer for bioreactor content analysis), at-line (manual sampling and analysis), and in-line (directly connected to the bioreactor) monitoring of samples, are near-infrared (NIR), fluorescence, IR, and Raman (Esmonde-White et al., 2017; Fisher et al., 2018). Raman spectroscopy has the ability to monitor structural/chemical changes in proteins. Glycoform patterns such as sialylation were directly observed on-line as a quality attribute criterion. Further development will be required to improve sensor design for easy integration into continuous bioprocessing systems (Fisher et al., 2018).

In a study by Kornecki and Strube (2018), *in-situ* turbidity and *ex-situ* Raman spectroscopy measurements were combined with an offline macroscopic Monod kinetic model in order to predict substrate concentrations in CHO cultivations in bioreactors. In another study, high-throughput MALDI mass spectrometry based on a microarray technology was used to observe N-glycopeptides of IgG1 produced in a perfusion cell culture (Hajduk et al., 2019). Different approaches for the determination of critical timepoints for product stability in an *E. coli* IB bioprocess were studied, and an empirical value was found that can be utilized as a process analytical tool (Slouka et al., 2019). An on-line method to control and manipulate glucose was studied and was validated to produce various recombinant therapeutic proteins across cell lines with different glucose consumption demands; it was then successfully demonstrated on micro (15 ml)-, laboratory (7 l)-, and pilot (50 l)-scale systems (Goldrick et al., 2018). For a *P*. *pastoris* fermentation to produce human interferon alpha 2b, a PAT platform was developed to monitor and control μ using capacitance (Δ*C*) during the induction phase (Katla et al., 2019). A novel approach based on the PAT initiative was also developed for on-line estimation of μ using *in-situ* dielectric spectroscopy (Li M. et al., 2019). The PAT framework was also used during the production of Lethal Toxin-Neutralizing Factor (LTNF) by *E. coli*, which was controlled by a decoupled input-output linearizing controller (DIOLC) (Dalal et al., 2019). LC-MS metabolomics at three bioreactor scales (10 l, 100 l, and 1,000 l) were utilized to gain insight into the basal metabolic states of the CHO cell culture during fed-batch, and this was demonstrated as a useful technique to obtain physiological information on the cell culture state during a bioprocess, regardless of scale (Vodopivec et al., 2019).

### Process Modeling

It remains a challenge to set up a universal mechanistic model for processes dealing with mammalian cells because of the lack of full knowledge of metabolic networks and reaction pathways. A hybrid semi-parametric model containing mechanistic and machine-learning methodologies has emerged as a potential tool for bioprocess development (Pinto J. et al., 2019). In one study, a mathematical model to describe polio virus production in batch bioreactors was developed and was able to accurately describe its production by Vero cells (Jiang Y. et al., 2019). The combination of mechanistic growth models with a parallel mini-bioreactor system for *E. coli* strain screening was studied to select the most robust strains with a scale-down approach for bioprocess scale-up (Anane et al., 2019). A hybrid model was studied using a 3.5 l fed-batch process for therapeutic protein production and was found to have a better capability to predict the time evolution of various process variables in comparison to statistical models (Narayanan et al., 2019b). A simple techno-economic model for mAbs production was also studied that can be used for any production platform (Mir-Artigues et al., 2019). Various other modeling approaches to optimize bioprocesses have also been studied (Gangadharan et al., 2019; Grilo and Mantalaris, 2019). A three-dimensional computational fluid dynamics (CFD) model was established for the analysis of the influence of baffle structure on the flow field in orbitally shaken bioreactors (OSRs), and it was proposed that the shear stress was gentle for mammalian cell growth (Zhu et al., 2019a). Further, a three-dimensional CFD model for hollow OSRs was established and validated, and it was verified that the hollow cylinder wall could improve the mixing efficiency (Zhu et al., 2019b). CFD simulations were also applied to analyze and compare microfluidic single-cell trapping and cultivation devices (Ho et al., 2019). In a study by Li et al. (2019), a scale-down model representing a 4,000-l culture process was established for foot and mouth disease vaccine production, and computational fluid dynamics (CFD) simulation was also used to study hydrodynamic environments inside the bioreactors.

### Perfusion Culture Process

The development of continuous perfusion bioreactor cultures provides cost and performance benefits to biopharmaceuticals producers (Somasundaram et al., 2018). This process continues for over a month or so, requiring an optimized process and cell-line stability to yield the largest amount of proteins. Perfusion bioreactor cultivations have also been utilized for seed bioreactors and cell stock preparations with a cell density of 1–2 × 10^8^ cells/ml (Clincke et al., 2013; Fisher et al., 2018). In a perfusion bioreactor, a harvest port and a bleed port are fitted to avoid the accumulation of toxic metabolites and to attain more viable cell density values (Karst et al., 2018).

Novel types of cell retention technique, such as alternating tangential flow (ATF) filtration, have been developed and used in perfusion bioreactors for recombinant protein manufacturing as well as the most commonly used TFF and spin filters (Rathore et al., 2015; Karst et al., 2016; Tapia et al., 2016; Bielser et al., 2018). In order to enhance the perfusion process yield, the bleed rate should be low (Lin et al., 2017). In ATF filtration, a diaphragm pump provides a flow of cell broth in alternating directions with a cycle time of about 1 min, and without additional shear stress and possible fouling (Gronemeyer et al., 2014; Patil and Walther, 2016; Hadpe et al., 2017). Karst et al. (2016) evaluated ATF vs. TFF and observed 50% retention in TFF as compared to 10% using ATF in the perfusion bioreactor. In another study, the use of ATF in place of an internal spin filter was studied, and it was found to result in higher cell densities, a higher perfusion rate, higher production (50–70%), and a longer run (Bosco et al., 2017). To overcome the product retention problem associated with TFF- and ATF-based perfusion cultures, a large pore size hollow fiber was recently used and drastically reduced product retention (Wang et al., 2019). In another study, temperature was established to be an important parameter in perfusion culture performance optimization (Wolf et al., 2019a). Comparative evaluation of fed-batch and perfusion platforms for IgG1- and IgG4-producing cell lines was carried out with ATF filtration, and perfusion was found to have 7.5 times greater average productivity (Walther et al., 2019). In another study, a two-step procedure [first, finding a suitable minimum value of the cell-specific perfusion rate (CSPR) at constant perfusion rate (P) or constant viable cell density (VCD) and second, investigating steady states at constant CSPR but elevated values of VCD and P] was developed for the design and development of CHO cell perfusion cultures and enabled high product yield and improved process performance with minor change in product quality (Wolf et al., 2019c).

Shake tubes (ST) were also established as an important scale-down tool for mammalian perfusion cell cultures in combination with bench-top bioreactors, giving high productivity (23 pg/cell·day) and low product loss in the bleed (around 10%) (Wolf et al., 2019b). A high-capacity microscale system for perfusion culture using *in-situ* gravity settling and automated sampling was studied, and the suitability of this platform for the evaluation of the performance of cell lines and the effects of process parameters for perfusion cultures were demonstrated (Sewell et al., 2019).

## Downstream Process Development

**Figure 2:**
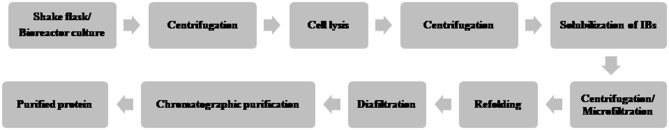
Flow chart of the purification of recombinant proteins expressed as IBs using *E. coli*.

The downstream purification process is of great importance, since it contributes to the approval of therapeutic products for human use (Owczarek et al., 2019). Conventionally, recombinant proteins are purified using centrifugation-, chromatography-, and membrane filtration-based purification steps. Apart from these steps, viral inactivation is also used for recombinant biopharmaceuticals produced using mammalian cells (Zydney, 2016). Cell disruption is required for recovery of the desired proteins, expressed as intracellular IBs (Ehgartner et al., 2017). The commonly used cell disruption methods at large scale include high-pressure homogenizers and bead mills (Mevada et al., 2019). A flow chart for the purification of IB-expressed recombinant protein is shown in Figure 2. This includes cell harvesting using centrifugation, cell lysis, IB solubilization, refolding, diafiltration, and chromatographic purification steps to obtain purified and biologically active protein.

Centrifugation, depth filtration, and tangential flow microfiltration (TFF-MF) are the most commonly used techniques for cell harvesting and cell separation (Besnard et al., 2016; Voulgaris et al., 2016; Richardson and Walker, 2018; Carvalho et al., 2019b; Yu et al., 2019). The various clarification technologies used for the downstream processing of antibodies have been described elsewhere (Singh et al., 2016; Singh and Chollangi, 2017).

**Figure 3:**
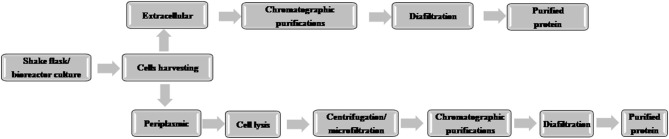
Flow chart of the purification of extracellular and periplasmic space-expressed recombinant proteins.

Refolding of proteins is necessary to attain biological activity for IB-expressed recombinant proteins. Batch mode refolding of solubilized IBs can be carried out using rapid or pulse dilution, by diafiltration and dialysis or by on-column chromatographic methods. In a recent study, it was also established that mild solubilization can be considered in terms of cost, time, and tag-free nature for the recovery of scFv from IBs (Sarker et al., 2019). The various methods used for refolding to recovery biologically active proteins have been reviewed previously (Rathore et al., 2013; Yamaguchi and Miyazaki, 2014). A flow chart tracking the purification of extracellular and periplasmic space-expressed recombinant proteins is shown in Figure 3. For extracellular-expressed recombinant proteins, post-cell harvesting purification steps include chromatography processes and diafiltration whereas, for periplasmic space-expressed recombinant proteins, the steps include cell lysis, centrifugation, microfiltration, chromatographic purifications, and diafiltration.

### Chromatography Processes

Various chromatography techniques, namely affinity, ion exchange, hydrophobic interaction, and size exclusion or gel filtration chromatography, are used to purify recombinant protein-based biopharmaceuticals to achieve a high purity product with a biologically active form (Saraswat et al., 2013; Rathore et al., 2018). It is well-established that an increased product concentration in the upstream process leads to a higher volume of chromatography resin and a higher buffer requirement. HCPs are the main source of impurities, and the HCPs of each process vary significantly from each other in their molecular mass, charge, hydrophobicity, and structure. Therefore, they present a challenge for chromatographic purification. It is possible to reduce the HCP production during upstream process development. Cell lines producing HCP at a lower level should be selected during upstream process development to aid efficient product purification (Gronemeyer et al., 2014).

Affinity chromatography (AC) is popular and is the most selective technique used for purification of tagged proteins, bispecific antibodies, DNA-based biologics, cellular products, viral vectors, and viruses (Zhao et al., 2018; Challener, 2019; Łacki and Riske, 2019). Some of the commonly used affinity tags are hexahistidine (His), glutathione S-transferase (GST), and maltose-binding protein (MBP) (Zhao et al., 2013). Protein A chromatography is the most widely used method for mAb purification (Gronemeyer et al., 2014; Sifniotis et al., 2019). The main problem associated with this chromatography is Protein A leachability, with non-specific binding of host cell protein, DNA, and other cell culture-derived impurities. Thus, it is necessary to recover these impurities using other chromatography techniques (Tarrant et al., 2012). In a recent review, challenges and progress in the purification of mAbs using Protein A chromatography were described, including elevated resin costs and their limited lifetime, Protein A ligand modification, and alternative formats such as monolith membranes and microspheres (Ramos-de-la-Peña et al., 2019).

Ion exchange chromatography is the most widely used and cost-effective method for the purification of recombinant proteins. Cation and anion exchange chromatography (CEX and AEX) remove various types of impurities such as product variants, remaining HCP and DNA, media components, leached Protein A, endotoxins, and viruses (Saraswat et al., 2013; Tripathi, 2016; Kimia et al., 2019). The efficacy of a weak anion exchanger on the isolation of rHBsAg VLPs from aggregated structures was also examined, and it was found to yield a 94–97.5% content of rHBsAg VLPs that were within the acceptable quality level (Kimia et al., 2019). Purification of rHBsAg derived from yeast crude extract was carried out using an AEX column, resulting in high purity (up to >95%) (Ashourian Moghadam et al., 2019). Viral safety is a critical concern for therapeutic proteins such as mAb produced using mammalian cells such as CHO cells. It was also reported that CEX carried out in overloaded mode was able to remove viruses during the manufacture of mAbs (Masuda et al., 2019). Hydrophobic interaction chromatography (HIC) is based on the relative hydrophobicity of the protein molecules. HIC is mostly utilized as a polishing step for the purification of recombinant proteins. In HIC, at high ionic strength, binding of proteins to ligands occurs whereas, at low ionic strength, elution of proteins occurs (McCue, 2009; Fekete et al., 2016). In a recent study, the influenza A and B viruses were successfully purified by HIC with up to 96% virus recoveries and about 1.3% residual DNA level using a 96-well-plate format (Weigel et al., 2019). Size exclusion chromatography (SEC) or gel filtration chromatography separates protein molecules based on their molecular weight. SEC is used for the purification of various proteins such as scFv and insulin-like growth factor receptor (Levin et al., 2015) as well as for aggregate removal and desalting (Brusotti et al., 2018; Burgess, 2018).

Membrane-based chromatography processes have also emerged as a good choice for recombinant protein purification. In this type of chromatography, a specific ligand is attached to microfiltration membrane pores. The impurities present in protein solutions bind to the membrane at neutral to slightly basic pH and low conductivity. The optimization parameters for protein purification using membrane-based chromatography include membrane size distribution and thickness and flow distribution (Orr et al., 2013; Boi and Dimartino, 2017; Gupta and Shukla, 2017c). Hydrogel and nanofiber base matrices of membranes offer a high specific area, higher ligand density, and an optimized 3D binding environment (Liu et al., 2017). It was also reported that the use of nanofibers within the membrane systems of affinity chromatography could represent a significant improvement over the current offerings for viral vector purification (Challener, 2019).

Due to the variable nature of the process and product-related impurities, it is not possible to purify a protein using single-step chromatography. Multimodal or mixed-mode chromatography involves selective interactions between the chromatography ligand and protein molecule through either ionic, hydrophobic, hydrogen bonding, or Van der Waals interactions. Mixed-mode resin can offer salt-tolerance, better separation, and higher binding capacity (Kallberg et al., 2012; Zhang and Liu, 2016; Halan et al., 2018; Sakhnini et al., 2019). The use of Capto resins (GE) is based on hydrophobic and ionic interactions, and that of Ceramic Hydroxyapatite (CHT) is based on electrostatic interaction and affinity interaction. MMC was successfully used as a capture step (using hydrophobic charge-induction chromatography) (Maria et al., 2015) and a polishing step (Bhambure et al., 2013). A Yellow Fever vaccine was prepared using bioreactors, followed by an anion exchange membrane adsorber, a multimodal resin, and β-propiolactone inactivation. The overall virus recovery in these chromatography steps was 52.7% (Pato et al., 2019). Recently, dextran-grafted mixed-mode chromatography adsorbents were prepared, which resulted in enhanced adsorption performance for BSA/IgG (Gu et al., 2019).

### Membrane Filtration-Based Techniques

Ultrafiltration is used for purification and protein concentration (Ledung et al., 2009; Emami et al., 2019; Palombarini et al., 2019). Diafiltration via an ultrafiltration membrane is used for desalting recombinant proteins (Kovács, 2016). The application of a filter aid (diatomaceous earth) coupled with crossflow ultrafiltration was also studied to remove contaminant proteins and DNA molecules without the use of chromatographic steps (Palombarini et al., 2019). In another study, ultrafiltration/diafiltration was used for the final purification of conjugated vaccine products (Emami et al., 2019). A filtration-based strategy consisting of a cascade of ultrafiltration and diafiltration steps followed by a sterile filtration step was also explored for the purification of influenza VLPs and achieved approximately 80% recoveries (Carvalho et al., 2019a). The combination of single-pass TFF concentration and AEX chromatography was also used for an intensified mAb polishing step that improved both manufacturing flexibility and process productivity (Elich et al., 2019). An efficient chromatin-directed clarification process for cell culture fluid, an alternative to Protein A chromatography, was developed for IgG purification to remove most host DNA and histones as well as to reduce non-histone HCPs. This allowed TFF to concentrate clarified supernatant and carry out buffer exchange, and cation exchange chromatography effectively removed the remaining host impurities to meet all clinical requirements (Liu W. et al., 2019b).

### Aqueous Two-Phase Extraction (ATPE), Precipitation, and Crystallization

Some other downstream processing steps, including aqueous two-phase extraction (ATPE), precipitation, and crystallization, were also used for recombinant protein preparation based on the requirements of various expression host systems (Hong Yang et al., 2013; Gronemeyer et al., 2014; Huettmann et al., 2014; Swartz et al., 2018; Andrade et al., 2019). Preparative protein crystallization was well-described in a recent review (Hubbuch et al., 2019).

## Recent Progress In Downstream Processing

Recent developments in downstream purification processes include the use of high-throughput devices, single-use systems, QbD and PAT, modeling, continuous downstream processing, and integrated continuous downstream processing.

### High-Throughput Technologies

High-throughput (HT) technologies have become an important aspect of downstream process development because of their potential to rapidly gather more data related to the process in comparison to traditional laboratory-scale techniques (Benner et al., 2019). For *E. coli*, HT-compatible bead mills were used for cell disruption (Lazarevic et al., 2013). Various other HT cell disruption/lysis devices have been used, including an 8-well-sonifier for VLPs from *E. coli*, a 24-well-HT sonication device for 15 cells including bacteria, fungi, and yeasts, and microfluidic channels (96-well-format) for thermal treatment, osmotic shocks, and freeze-drying (Baumann and Hubbuch, 2017). In a review, microscale disruption of microorganisms (as low as 200 μl) for parallelized process development was discussed in detail along with their performance compared with high-pressure homogenization (Walther and Dürauer, 2017). HT refolding systems for IB-expressed proteins are also available commercially and are listed in Table 5 (Baumann and Hubbuch, 2017), together with some of the other HTP devices used in downstream process development.

**Table 5:** List of some HTPD systems used for downstream process development (Baumann and Hubbuch, 2017).

**System details**	**Manufacturer/developer**
Chromafil Multi 96-filter plates (0.2, 0.45, 1, 3, 20, and 50 μm) (for simultaneous filtration of 96 samples; different filter materials)	Macherey–Nagel
PD MultiTrap G-25 (96-well-plates; desalting and buffer exchange; 0.5 ml)	GE
PreDictor Plates for chromatography (96-well-pre-packed filter plates; 6–50 μl)	
HiTrap Columns for column chromatography (1 or 5 ml)	
Pierce 96-well-Microdialysis Plate, 10 kDa MWCO (12 cartridges of 8 microdialysis devices; 10–100 μl sample volume)	Thermo Fisher
Pro-Matrix™ Protein Refolding Kit for 100 refolding reactions	
PhyNexus Phy Tip columns for chromatography (filter pipet tips pre-packed with resins)	PhyNexus
AcroPrep Advance 96-well-filter plate for ultrafiltration	Pall
AcroPrep Advance 96-well-filter plate for chromatography	
AcroSep Chromatography Columns (1 ml)	
MediaScout ResiQuot for batch chromatography (8 or 20 μl)	ATOLL
MediaScout MiniChrom for column chromatography (0.2–10 ml)	
MediaScout RoboColumn for column chromatography (0.2 or 0.6 ml)	

HT chromatography systems with different capacities are available (Rege et al., 2006; Coffman et al., 2008; Chhatre and Titchener-Hooker, 2009; Lacki and Brekkan, 2011; Łacki, 2012; Chu et al., 2018). Bind and elute evaluations for mAbs and amyloglucosidase have been carried out using pre-packed PreDictor filter plates (GE). Other HT devices have been used successfully, such as AcroPrep Advance 96-well-filter plates (Pall) for the G-CSF, PhyNexus tips (PhyNexus) for Fab fragments, MediaScout MiniChrom columns (Atoll) for mAbs, MediaScout RoboColumn (Atoll) (200–600 μL columns) for mAb and antibody fragments, and HiTrap columns (GE) for recombinant HIV-1 capsid protein purification (Urmann et al., 2010; Treier et al., 2012; Hung et al., 2013; Muthukumar and Rathore, 2013; Brenac Brochier and Ravault, 2016; Baumann and Hubbuch, 2017). In a recent study, the pairing of MiniColumns and Tecan liquid handlers was used to run up to eight chromatography conditions in one experiment (Benner et al., 2019). An automated HT batch-binding screen using a 96-well-filter-plate (Seahorse Bio) for CEX resins was efficiently optimized for step elution to increase purity and yield for antibodies (McDonald et al., 2016). An HT method based on a microtiter filter plate [96-well with MultiScreen_HTS_ Vacuum Manifold (Merck-Millipore)] was applied to determine the adsorption properties and evaluate the optimal conditions for human serum albumin (HSA) isolation with four MM resins and two IEX resins; the findings were verified by laboratory-scale column chromatography (Chu et al., 2018). In another study, an HT process development workflow integrated with a microscale chromatography, DoE, and multivariate data analysis was studied and provided a rational method for screening resins and process parameters (Stamatis et al., 2019). In a study by Andar et al. (2019), a microscale column using IMAC was used for the purification of G-CSF expressed using a cell-free CHO and was compared with a 1 ml IMAC column. A 10-fold decrease in buffer, resin, and time of purification was observed in comparison to conventional columns for similar protein yields. In a recent study, using 96-well-plates containing nickel-functionalized membranes, rapid screening of parameters for membrane protein purification was successfully performed (Feroz et al., 2019). Mixed-mode resins (ionic and hydrophobic interactions) were used in a plate-based HT screening platform for the selection of process parameters to achieve high purity and high overall yield of osteopontin (Guo et al., 2019). A novel microfluidics-based methodology to carry out speedy and multiplexed screening of several MM ligands relative to their potential to bind different target molecules was studied using an artificial mixture (containing IgG and BSA, labeled with different thiol-reactive neutral fluorescent dyes). The study report suggested that this strategy can potentially be utilized as a predictive analytical tool in the context of purification of mAb (Pinto I. F. et al., 2019). The benefits of a HT chromatography system include its availability to predict design space for dynamic binding capacity (DBC), collect data for the prediction of elution behavior, and allow significant investigation using DoE (McDonald et al., 2016). HT chromatography systems have limitations in their potential to reveal the flow distribution of process columns (Singh and Herzer, 2017).

Noyes et al. (2015) studied an ultra scale-down device for high-throughput depth filtration that enabled the parallel assessment of eight single- or multi-layer depth filters (~0.2 cm^2^ in cross-sectional area). In another study, a novel HT filtration screening system was used to characterize the proteins of different feedstreams with antibody concentrations of up to 20 g/l for their viral filtration performance using either low-interacting or hydrophobically interacting pre-filters. This study indicated the existence of two different fouling mechanisms: an irreversible and a reversible mechanism (Bieberbach et al., 2019). The performance of a pilot-scale TFF system was predicted by devising an ultra scale-down (USD) device consisting of a cell stirred using a rotating disc (2.1 cm^2^ of membrane area and 1.7 ml of feed), with good agreement between the USD and TFF devices in terms of the flux and resistance values for a mAb diafiltration stage (Fernandez-Cerezo et al., 2019).

Aqueous two-phase extraction has the potential to selectively separate proteins from unclarified cell culture supernatants directly. In one study, microfluidic aqueous two-phase extraction screening systems with fluorescence microscopy were demonstrated, and it was reported that the partition coefficient (K_p_) measured in PEG 3350–phosphate systems with and without the addition of NaCl using microtubes (batch) or microfluidic devices (continuous) was similar to those calculated for the native protein (São Pedro et al., 2019).

### Single-Use Technologies

For single-use or disposable cell harvesting, two main options, namely a single-use centrifuge followed by single-use depth filters or single-use depth filters alone, are used. Single-use depth filters are more common due to the commercial unavailability of single-use large-scale centrifuges (Boedeker et al., 2017). For centrifugation, kSep^®^ single-use continuous centrifuges (kSep400 and kSep6000S) (Sartorius) were developed and successfully used for cell harvesting to purify recombinant proteins (Mehta, 2014). The Unifuge is another single-use centrifuge available for cell harvesting. Due to the development of single-use filtration techniques, primary and secondary filters can be replaced by a single filtration step. This leads to a lower cycle time, filtration surface area, and buffer requirement. Depth filters can be employed to recover cells from single-use bioreactors up to the 2,000 l scale. However, the number of systems needed for a 2,000 l bioreactor culture is greater, so their use should be analyzed with respect to cost, space, waste, footprint, etc. (Boedeker et al., 2017). The disposable depth filters Stax (Pall), Clarisolve, and Millistak D0HC and X0HC (Merck-Millipore) are available and are used for efficient cell clarification (Schreffler et al., 2015). The depth filters have advantages like ease of scalability, better recovery, consistency, and low cost (Collins and Levison, 2016). However, some issues associated with single-use depth filters include the binding of proteins or DNA (Gupta and Shukla, 2017c).

Single-use chromatography systems can be utilized for the purification of recombinant proteins from culture harvested from an up to 2,000 l bioreactor, depending on product titers, the loading capacity of the column, and process flow rates. Such a system is supported by pinch valves, sensors, and pumps (Boedeker et al., 2017). In a previous study using Protein-A, mixed-mode, and AEX resin columns, single-use continuous purification of mAb was achieved using AKTA periodic counter-current chromatography (Mothes, 2017). It was reported that the per gram mAb operating cost of an SU facility is 22% lower than that of a stainless steel (SS) facility (Gupta and Shukla, 2017c). Single-use TFF systems (Pall and Merck-Millipore) are commercially available for downstream purification, and these systems consist of pumps, pinch valves, a tank, sensors, and tubing manifolds (Boedeker et al., 2017).

### Design of Experiments (DoE) Approach

The use of DoE has also been established to increase the performance of downstream process development. In one study, high-pressure homogenization was used to screen critical process parameters (CPPs) using DoE to enhance product titer and achieve adequate product quality, based on predefined critical quality attributes (CQAs) (Pekarsky et al., 2019). A process for the purification of scFv using mixed-mode chromatography was developed using DoE, and it was found that the optimized conditions enabled binding of the scFv to Capto Adhere™ below its theoretical pI, with the majority of HCPs in the flow-through (Sakhnini et al., 2019). A split DOE approach was successfully used in HIC to remove aggregates, and CEX was used to isolate charge variants and aggregates, resulting in a reduction of the total number of experiments by 25 and 72% compared to a single DoE based on CCD and FFD, respectively (Shekhawat et al., 2019).

### Process Analytical Technology (PAT) for Downstream Processing

In downstream processing, PAT tools are used for the analysis of protein concentration, its purity, host cell proteins, host cell DNA, endotoxin, variants (misfolding), and process-related impurities. For these purposes, spectroscopy, spectrometry, HPLC, circular dichroism, and other tools are used to monitor critical quality attributes in chromatography processes. Next-generation sequencing could be used for virus screening, but it is very sophisticated. Further studies are needed to determine the critical points to assure the viral safety of therapeutic proteins (Fisher et al., 2018). One study used an on-line HPLC as a PAT tool for automated sampling of a product stream eluting from a chromatography process column (Tiwari et al., 2018). FTIR spectroscopy as a PAT tool was also used for near real time in-line estimation of the degree of PEGylation in chromatography (Sanden et al., 2019). At-line multi-angle light scattering and fluorescence detectors were used in the downstream processing of HEK293 cell-produced enveloped VLPs containing the HIV-1 Gag protein fused to the Green Fluorescence protein (Aguilar et al., 2019).

### Modeling Approach

Modeling and simulations can significantly decrease the number of experiments needed while increasing or collecting experimental data (Hanke and Ottens, 2014). Empirical models are based on a priori identified output data within a defined design space, and mechanistic models are based on physicochemical properties (Baumann and Hubbuch, 2017). Mechanistic modeling is an important process development tool that has been used for chromatography to speed up process development. These models can explain the downstream unit operation at a level of detail that depends on the application (Benner et al., 2019). The use of a mechanistic model of HIC as a PAT tool for pooling decisions to enable aggregate removal for a mAb resulted in higher product purity with respect to offline column fractionation-based pooling (Shekhawat and Rathore, 2019b). An approach toward statistical process control and monitoring of protein refolding during the production of recombinant therapeutic proteins from *E. coli* was described in a study by Hebbi et al. (2019). This approach used on-line measurements of redox potential, temperature, and pH for the development of a statistical model. This was successfully demonstrated to ensure the quality of the manufactured product consistently. An empirical interpolation (EI) method was used to predict elution performance on a CEX column based on batch isotherm data and revealed good agreement with experimental elution curves for the separation of mAb monomer and dimer mixtures for protein loads up to 40 mg/ml column or about 50% of the column binding capacity (Creasy et al., 2019). Early-stage bioprocess development faces the issues of the definition of optimal operating parameters. Polishing chromatography of a mAb from a challenging ternary feed mixture was optimized by a hybrid approach of the simplex method and a form of local optimization. The findings of the study showed it to be perfectly suitable for the speedy development of bioprocessing unit operations (Fischer et al., 2019). An overview of mechanistic modeling of liquid chromatography was given in a recent study (Shekhawat and Rathore, 2019a).

### Continuous Downstream Processing

**Figure 4:**
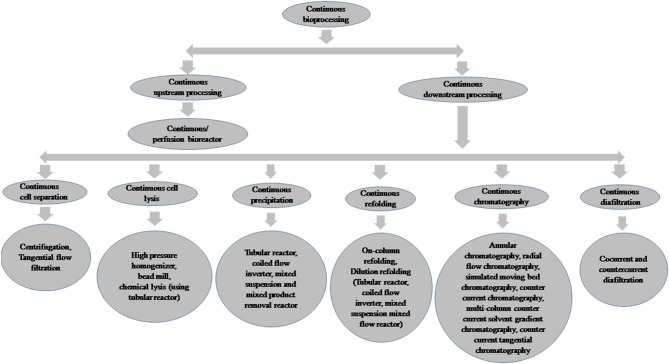
Flow chart of the different technologies within continuous bioprocessing.

The shift from a traditional batch process to a continuous process for any product can reduce cost (Schofield, 2018). Systems and techniques for continuous downstream processing of biopharmaceuticals have been developed and used for process development and scale-up. The various technologies of continuous bioprocessing are shown in Figure 4. Continuous centrifugation and TFF-MF are the main methods utilized for cell harvesting or cell removal. Disk stack and tubular bowl centrifuges have been used in continuous operation for the harvesting of a recombinant *E. coli* fermentation that was carried out for a domain antibody production (Voulgaris et al., 2016). A disk-stack continuous centrifuge with periodic and continuous discharge was also used for large-scale clarification of high cell density CHO cell culture for IgG1 mAb production (Richardson and Walker, 2018). The cell lysis techniques (mechanical type) used in continuous mode are high-pressure homogenization and bead milling. In a study by Haque et al. (2016), continuous bead milling was used for the recovery of a recombinant protein, and the process was optimized using RSM.

### Continuous Precipitation

In the continuous mode of precipitation, a stirred tank reactor, MSMPR, tubular reactor, and centrifugal precipitation chromatography can mainly be used for bioproducts. A continuous precipitation process for mAbs using a tubular reactor was studied with PEG and resulted in 86–94% yields with HCP reduction (7,200–15,000 ppm) (Hammerschmidt et al., 2016). A coiled flow inverter reactor has also been used for continuous precipitation of clarified cell culture supernatant based on pH, CaCl_2_, and caprylic acid and resulted in comparable or increased productivity vs. a batch process (Kateja et al., 2016). A combination of reversible cross-linking (ZnCl_2_) and volume exclusion (polyethylene glycol) agents was also established to continuously precipitate a mAb product in a tubular reactor directly from clarified cell culture fluid (CCCF) (Li Z. et al., 2019).

### Continuous Aqueous Two-Phase Extraction

Aqueous two phase extraction in continuous mode can be carried out by column contactors, mixer-settler, spray columns, and rotating disk contactors (Eggersgluess et al., 2014; Espitia-Saloma et al., 2014). A continuous ATPE system for human IgG in a microfluidic device (mixer-settler) in one-stage, multistage, and multistage with recirculation setup was studied with a PEG-3350 phosphate ATPS and resulted in 65 and 90% recovery with one-stage and multistage, respectively, along with 78% in recirculation (Espitia-Saloma et al., 2016).

### Continuous Chromatography

In continuous downstream processing, continuous chromatography processes are crucial to achieve high purity for proteins, and these processes are in an advanced stage, with a variety of options. By operating many chromatography columns in a countercurrent or concurrent manner, continuous operation can be achieved, as the loading is carried out in the first column and all of the other steps (washing, elution, regeneration, and re-equilibration) in the subsequent ones (Jungbauer, 2013). Continuous annular chromatography (CAC), simulated moving bed (SMB) chromatography, countercurrent chromatography (CCC), multicolumn countercurrent solvent gradient purification chromatography (MCSGP), and countercurrent tangential (CCT) chromatography are used in the continuous mode of operation (Pagkaliwangan et al., 2018; Rathore et al., 2018; Vogg et al., 2018). Some of the commercially available continuous chromatography platforms are listed in Table 6. One study performing a comparative cost analysis of batch vs. continuous process for 200 kg mAb (annual production) showed that the latter resulted in a decrease in the downstream processing cost of goods (COGs) by ~8€/g of mAb, with increased requirements of culture medium (Klutz et al., 2016; Somasundaram et al., 2018).

**Table 6:** List of some commercially available continuous chromatography systems used for recombinant biopharmaceuticals.

**System details**	**Feed**	**Applications**	**Manufacturer/developer**
3C-PCC/4C- PCC (3 or 4 columns)	Continuous	Protein A, HIC, CEX, MMC	GE
Step SMB (8 columns)	Continuous	Protein A, SEC	Semba
BioSMB (3–16 columns)	Continuous	Protein A, CEX	Pall
BioSC SMCC (2–6 columns)	Semi-continuous	Protein A	Novasep
Capture SMB (2-column)	Semi-continuous	Protein A	ChromaCon

MCSGP with a four-column system was successfully utilized for the initial capture of an IgG2 mAb from CCCF using CEX with gradient elution (Müller-Späth et al., 2010). A study of the application of CCT chromatography using Protein A resin for the initial capture and purification of two commercial mAbs from CCCF showed that it resulted in similar characteristics in terms of HCP removal, product yield, and purity as conventional column chromatography (Dutta et al., 2015). The use of continuous capture multi-column chromatography (BioSMB) at laboratory scale for a mAb capture process using Protein A resin was successfully validated (Gjoka et al., 2015). In a study on mAb capture using Protein A-based twin-column CaptureSMB, it was reported that the resin cost could be reduced by up to 10–30% (Angarita et al., 2015). Twin-column CaptureSMB and three and four-column PCC were studied for capturing mAb using Protein A resin and resulted in similar maximum capacity utilization (Baur et al., 2016). An integrated two-stage chromatographic process platform containing CEX and MM was used for the separation of charge variants and aggregates for three different mAbs, and it was found that the required aggregate (<1%), HCP (<10 ppm), and DNA (<5 ppb) clearance was achieved (Kateja et al., 2017b). CCT chromatography was also used for a post-capture antibody purification step using MM resins (CEX-HIC) and showed a 5% increase in yield with similar contaminant removal (Dutta et al., 2017). In another study, it was established that the chromatography resin in a two-column continuous system resulted in 2.5-fold more utilization in comparison with single column batch system (Steinebach et al., 2016; Bielser et al., 2018).

In a scale-up study for purification of mAbs, it was reported that buffer savings of around 50% were achieved using a PCC strategy (Angelo et al., 2018). Four different loading scenarios with a Cadence BioSMB MCC for the Protein A mAb capture step were evaluated, and it was concluded that by adding more columns, up to 65% more productivity (at feed concentrations of above 5 g/l) could be achieved (Pagkaliwangan et al., 2018). The effect of particle size (85 vs. 50 μm) on the performance of continuous capture Protein A affinity chromatography was studied with respect to feed titers, load flow rates, and target breakthrough with single column batch, two-column CaptureSMB, and four-column PCC using a DOE approach. The 50 μm resin resulted in better productivity as compared to the 85 μm resin (Baur et al., 2018). The impact of two different quality feeds (one from depth filtration and other from a combination of depth filtration and chromatographic clarification) on Protein A PCC was studied, with the result that there was 49% increased productivity for the chromatographically clarified material over 100 cycles, with 11-fold lower HCP and a 4.4 LRV for HCDNA (El-Sabbahy et al., 2018). Upscaling of Protein A continuous chromatography using the Cadence™ BioSMB PD and the Cadence™ BioSMB Process 80 system was successfully carried out for a 10-day run time using feed from a perfusion culture and resulted in a 400–500% increase in vs. batch mode (Ötes et al., 2018). In another study, recovery, and enrichment of the native form of an mAb and of basic and acidic variants were achieved in a multi-column continuous chromatography set-up (three-column) by self-displacement chromatography with a process yield of over 90% (Khanal et al., 2019). In an MCSGP process, by means of the isolation of the main charge isoform of an antibody, the purity was determined by the selection of the product collection window, with negligible influence from the recycle phases (Vogg et al., 2019).

One study considered “standard,” “model-assisted,” and “hybrid” approaches to process characterization for validating continuous twin-column capture chromatography (CaptureSMB) with CCCF containing an IgG_4_ at 5 g/l (Baur et al., 2019). Methods for the purification of human mAb and their fragments using different chromatography techniques, including continuous chromatography, were also described in a recent study (Ulmer et al., 2019b). A DoE approach using a single column (batch mode) was studied to simulate a multi-column (continuous mode) purification strategy with Protein A capture, anion exchange, and MM cation exchange, and robust and predictable continuous bioprocesses were developed. The process developed yielded total product recovery at or above 74%, HCP (<5 ppm) and an aggregate content below 1% (Utturkar et al., 2019).

### Continuous Viral Inactivation and Clearance

In the case of manufacturing therapeutics using CHO cells, viral clearance is mandatory (Chiang et al., 2019; Jungbauer, 2019). Viral clearance for mAbs production processes uses a low pH hold because the protein elution occurs at low pH from a Protein A chromatography column. Continuous viral inactivation using a tubular reactor with a static mixer, a coiled flow inverter reactor, and a four-valve system with a mixer has been studied. A fully automatic Cadence™ (Pall) low-pH continuous viral inactivation system was developed and used for virus inactivation (Johnson et al., 2017; Gillespie et al., 2018). A packed-bed continuous viral inactivation reactor was used for the inactivation of two commonly used model viruses with a very low pressure drop and scalability (Martins et al., 2019). In a recent study, a coiled flow inverter was used for continuous low pH viral inactivation, and complete viral inactivation was achieved within the first 14.5 min for both continuous and batch studies (David et al., 2019).

### Continuous Refolding

Progress has also been made in the continuous refolding process. A coiled flow inverter reactor, packed column plug flow reactor (incorporated with a mixing system), a CSTR connected with a diafiltration system (for buffer exchange), continuous chromatography systems, or a tubular reactor can be used for continuous refolding. In one study, integrated continuous matrix-assisted refolding and purification by tandem SMB SEC was successfully achieved for Npro fusion proteins expressed in IBs (Wellhoefer et al., 2014). An integrated continuous tubular reactor system was utilized for continuous dissolving, refolding, and precipitation (Pan et al., 2014).

### Continuous Formulation

Diafiltration is mainly used for desalting and buffer exchange using ultrafiltration membranes. The co-current and countercurrent modes are used for continuous diafiltration (Kovács, 2016). In a study by Rucker-Pezzini et al. (2018), continuous three-stage single-pass diafiltration was studied and resulted in buffer exchange of >99.75%. A countercurrent staged diafiltration process was performed for continuous protein formulation for a polyclonal IgG with Cadence™ Inline concentrators (Nambiar et al., 2017). Cadence™ in-line concentrators (Delta 30 kDa membranes) were used in the three stages to obtain high conversion in a single pass and provided important insights into the design and operation of a continuous process for antibody formulation (Jabra et al., 2019). Countercurrent dialysis for continuous protein formulation and buffer exchange was done using concentrated solutions of IgG with commercially available hollow fiber dialyzers (1.5 and 1.8 m^2^ membrane surface area) (Yehl et al., 2019).

Crystallization for protein formulations can be carried out in continuous mode (Hekmat, 2015; dos Santos et al., 2017; Van Alstine and Łacki, 2018). In one study, continuous crystallization of a full-length therapeutic mAb was carried out using a laboratory-scale stirred tank (with a cooled tubular reactor in bypass) and resulted in a space–time yield of up to 12 g/l.h (Hekmat et al., 2017). Approaches to and the scientific understanding of controls over the crystallization–purification process in continuous crystallization were recently described in a review (Darmali et al., 2019).

A novel concept for the freeze-drying of pharmaceutics in unit-doses was presented by Capozzi et al. (2019), who reported that this configuration made it possible to set up a continuous freeze-drying process.

## Integrated Continuous Bioprocessing

Integrated continuous bioprocessing is currently gaining importance due to competition over product stability and cost as well as the large number of products available in the pipeline as compared to the low current facility capacity. Production and quality-related problems are the causes of almost two-thirds of all biological drug shortages. To overcome these problems, there is an increased trend across biopharmaceutical manufacturing toward process intensification and continuous production of biopharmaceuticals. Advances in bioprocessing technology have the capability to reduce shortages and variability, permit for manufacturing flexibility, simplify scale-up methodologies, reduce facility footprints and capital costs, enhance product yield, and decrease production costs (Fisher et al., 2018). It is also clear that a proper real-time monitoring and control system such as SCADA is needed to operate the whole process as one unit (Karst et al., 2018). PAT tools with a control system were successfully used to continuously measure the product titer at bioreactor discharge in a continuous integrated bioprocess carried out using a perfusion bioreactor with CaptureSMB Protein A chromatography (Karst et al., 2017, 2018).

Integrated continuous bioprocessing was used for manufacturing a drug substance that comprised a bioreactor with an ATF cell retention system and two PCC columns [one for capture (Protein A) and other for polishing (CEX)] including a viral inactivation step. This process resulted in a productivity of more than 600 g/l resin/day (Godawat et al., 2015). An integrated continuous downstream process (from IBs to an unformulated drug substance) consisting of a coiled flow inverter reactor for refolding, a three-column PCC for protein capture, and three-column concurrent chromatography for product polishing was used for a therapeutic protein (G-CSF) and achieved more than 99% purity with more resin utilization (Kateja et al., 2017a).

The design and operation of an integrated continuous bioprocess comprising of continuous cultivation with ATF, a continuous twin-column capture chromatography step, viral inactivation, a semi-continuous polishing chromatography step (twin-column MCSGP), and a batch flow-through polishing chromatography step was studied for continuous production of a commercial mAb. This process resulted in steady operation and uniform product quality over the 17 cycles of the end-to-end integration (Steinebach et al., 2017). A continuous integrated downstream process in which Protein A chromatography (capture), viral inactivation, flow-through anion exchange, and MM cation exchange chromatography were integrated across two Cadence BioSMB PD multi-column chromatography systems to purify a 25 l volume of harvested CCF. This process resulted in increased productivity and reduced resin and buffer requirements compared to a batch process (Gjoka et al., 2017). In another study, an integrated continuous biomanufacturing process using perfusion bioreactor culture with ATF and one-column continuous chromatography (OCC) was used to produce therapeutic mAbs and achieved an 80% enhancement in productivity (Kamga et al., 2018).

A fully integrated continuous bioprocess comprising a perfusion bioreactor with ATF, multicolumn chromatography, viral inactivation, depth filtration, single-pass TFF, AEX membrane polishing, viral filtration, and single-pass UFDF was used for mAb drug substance production. Comparable product quality with 4.6-times enhanced productivity was obtained in comparison to a fed-batch process. Further evaluation also revealed that a fed-batch facility (4 × 12,500 l SS bioreactors) and purification train of the corresponding scale could be substituted by a continuous facility (5 × 2,000 l SU bioreactors) and a smaller purification train, affording a 15% cost reduction (Arnold et al., 2018).

An integrated continuous bioprocessing platform containing a coiled flow inverter reactor for protein precipitation, protein capture using CEX, polishing steps using MM chromatography, and a salt-tolerant AEX membrane has been used for three different mAbs, and the process continued for 48 h using 1.4 l of CCF. In all scenarios, an acceptable process yield was achieved (70–80%), with consistent final-product quality attributes (Kateja et al., 2018). In another study, a process to intensify the enzymatic digestion of IgG and the purification of the resultant Fab fragment was established. The process consisted of the integration of a continuous packed-bed reactor into a novel multi-column countercurrent solvent gradient purification (MCSGP) process (by adding a third column to the classical two-column MCSGP process) (Ulmer et al., 2019a).

In a study by Yousefipour et al. (2019), an integrated system consisting of SEC and ultracentrifugation was used for the purification of recombinant hepatitis B surface antigen and achieved a 95% removal of protein impurities. A continuous precipitation process (PEG6000 and Zn^++^) in a tubular reactor integrated with a two-stage continuous TFF unit was also used and was reported to achieve 97% antibody purity and a 95% process yield during continuous operation (Burgstaller et al., 2019).

Though there has been tremendous progress in integrated continuous bioprocessing, many challenges are also associated with this process for therapeutic proteins. Upstream processes require strict sterility, but many downstream processes do not require sterility. Therefore, a sterile barrier is needed between upstream and downstream processes for integrated bioprocessing. Since both upstream and downstream processing systems have been developed independently, there is a lack of synchronization between them. Feedback control systems need to be developed, because various upstream parameters (e.g., HCP) influence downstream operations (e.g., purification) (Fisher et al., 2018). PAT tool innovations, such as the use of Circular Dichroism (CD) for the analysis of protein folding and qPCR for viral contamination, among others, are required to bring the finished product within regulatory requirements. It is also challenging to select and develop continuous cell removal and continuous cell lysis techniques appropriate to various expression systems. Various other issues associated with the development of effective integrated continuous bioprocessing are equipment robustness and operability, cell line stability, process sterility, viral contamination risk, process control, ability to scale up, validation, product stability, startup and shutdown, regulatory requirements, process time and operation cost, product concentration, product quality, and accumulation of waste product. All of these issues have to be considered for the development of effective integrated bioprocessing for the successful production of therapeutic proteins.

## Conclusion

The manufacturing of recombinant therapeutic proteins is a complex, multidisciplinary, and costly process. The demand for recombinant proteins for human application is increasing day by day. There is a huge demand for novel and improved bioprocessing strategies that are cost-effective and time-saving. The continuous improvement in biopharmaceutical expression systems has led to the production of quality products. Modern molecular biology techniques are at the forefront of the production of biopharmaceutical proteins using various prokaryote or eukaryote expression systems. Various innovative techniques, namely systems biology, metabolic engineering, and CRISPR/Cas systems, can be applied for strain engineering to improve bioprocess performance and to generate biologically active and stable proteins. Glycoengineering strategies may allow the easy production of a therapeutic protein with improved biological activity and safety. HTPD, single-use systems, and continuous bioprocessing are seen as enormously important developments. Single-use systems are increasingly used in both upstream and downstream process development, increasing the flexibility and production rate along with reducing capital cost and downtime. In spite of many developments in integrated continuous biomanufacturing and in single-use systems, there are various components that need further development, e.g., the integration of hardware and software. Truly continuous separation technologies in place of semi-continuous one will also help in the advancement of continuous bioprocessing, such as in cases like continuous chromatography and viral inactivation. The use of continuous bioprocessing for the production of biopharmaceuticals could reduce facilities and equipment footprint and capital and labor cost. Although many innovations have occurred in the area of continuous bioprocessing, fully synchronized upstream and downstream processing is still lacking. A well-balanced and systematic approach to continuous upstream and continuous downstream processing along with process and product characterization will realize a fully end-to-end continuous integrated bioprocess for biopharmaceuticals. Approaches for the quality assurance of the therapeutics are continuously evolving. The QbD strategy is recommended by regulatory bodies for a steady process and better-quality protein production. The use of advanced process analytical technology for direct and real-time analysis of critical product quality attributes like product concentration and contaminants during the operation and at discharge will play a major part in the success of bioprocessing and also fulfill the regulatory requirements. In future, thorough research is required, giving attention to the integration of various bioprocessing steps into a single operation and the optimization of the end-to-end process as a whole. Biopharmaceutical manufacturers are continuing to move toward more simple, robust, and automatic platforms and cost-effective product development, which can support the development of economical processes and inexpensive therapeutic development for a large population.

## Author Contributions

All authors listed have made a substantial, direct and intellectual contribution to the work, and approved it for publication.

### Conflict of Interest

The authors declare that the research was conducted in the absence of any commercial or financial relationships that could be construed as a potential conflict of interest.
